# Nanobodies Protecting From Lethal SARS-CoV-2 Infection Target Receptor Binding Epitopes Preserved in Virus Variants Other Than Omicron

**DOI:** 10.3389/fimmu.2022.863831

**Published:** 2022-04-25

**Authors:** José M. Casasnovas, Yago Margolles, María A. Noriega, María Guzmán, Rocío Arranz, Roberto Melero, Mercedes Casanova, Juan Alberto Corbera, Nereida Jiménez-de-Oya, Pablo Gastaminza, Urtzi Garaigorta, Juan Carlos Saiz, Miguel Ángel Martín-Acebes, Luis Ángel Fernández

**Affiliations:** ^1^ Departments of Macromolecule Structure, Microbial Biotechnology, and Cellular and Molecular Biology, Centro Nacional de Biotecnología, Consejo Superior de Investigaciones Científicas (CNB-CSIC), Madrid, Spain; ^2^ Instituto Universitario de Investigaciones Biomédicas y Sanitarias (IUIBS), Facultad de Veterinaria, Universidad de Las Palmas de Gran Canaria (ULPGC), Campus Universitario de Arucas, Arucas, Spain; ^3^ Department of Biotechnology, Instituto Nacional de Investigación y Tecnología Agraria y Alimentaria, Consejo Superior de Investigaciones Científicas (INIA, CSIC), Madrid, Spain

**Keywords:** coronavirus, COVID-19, nanobodies, neutralizing antibodies, SARS-CoV-2 variants

## Abstract

The emergence of SARS-CoV-2 variants that escape from immune neutralization are challenging vaccines and antibodies developed to stop the COVID-19 pandemic. Thus, it is important to establish therapeutics directed toward multiple or specific SARS-CoV-2 variants. The envelope spike (S) glycoprotein of SARS-CoV-2 is the key target of neutralizing antibodies (Abs). We selected a panel of nine nanobodies (Nbs) from dromedary camels immunized with the receptor-binding domain (RBD) of the S, and engineered Nb fusions as humanized heavy chain Abs (hcAbs). Nbs and derived hcAbs bound with subnanomolar or picomolar affinities to the S and its RBD, and S-binding cross-competition clustered them in two different groups. Most of the hcAbs hindered RBD binding to its human ACE2 (hACE2) receptor, blocked cell entry of viruses pseudotyped with the S protein and neutralized SARS-CoV-2 infection in cell cultures. Four potent neutralizing hcAbs prevented the progression to lethal SARS-CoV-2 infection in hACE2-transgenic mice, demonstrating their therapeutic potential. Cryo-electron microscopy identified Nb binding epitopes in and out the receptor binding motif (RBM), and showed different ways to prevent virus binding to its cell entry receptor. The Nb binding modes were consistent with its recognition of SARS-CoV-2 RBD variants; mono and bispecific hcAbs efficiently bound all variants of concern except omicron, which emphasized the immune escape capacity of this latest variant.

## Introduction

The COVID-19 pandemic is caused by severe acute respiratory syndrome coronavirus 2 (SARS-CoV-2) (1), and is a major threat to global public health that has caused over 6 million deaths (https://coronavirus.jhu.edu/) because of the absence of specific therapeutics. During last year, several COVID-19 vaccines based on different technologies were authorized in different countries, as well as some SARS-CoV-2 neutralizing Abs generated from COVID-convalescent individuals and humanized mice (2, 3). Nonetheless, these therapies are being challenged by the emergence of virus variants with enhanced transmission that scape from immune neutralization, which likely requires the update of vaccines and therapeutic Abs.

Most of the Abs that neutralize viruses, such as the SARS-CoV-2, bind to exposed proteins on the virus particle, and prevent virus cell entry, the initial step in the viral life cycle. In CoV, the virus envelope spike (S) glycoprotein is used for virus attachment to host cells and membrane fusion (4), and it is the main target of neutralizing Abs (3). The SARS-CoV-2 S forms a trimer on the virion surface (5), and it is cleaved during virus assembly into the N-terminal S1 and the membrane-bound S2 regions, which confers greater infectivity to this CoV (6). Additional S cleavage at the target cell surface primes SARS-CoV-2 cell entry (7). Several structures have defined the S domain architecture and identified two conformations (closed and open) for its receptor-binding domain (RBD), which locates at the C-terminal portion of the S1 region (8, 9). The RBD in the open conformation engages the SARS-CoV-2 entry receptor on the host cell surface, angiotensin-converting enzyme 2 (ACE2), which triggers S protein rearrangements that lead to membrane fusion and virus penetration into the cell (10). The SARS-CoV-2 RBD spans S residues 330 to 527 and is composed of two subdomains (11), the core, with a central β-sheet, and the external subdomain or receptor binding motif (RBM, residues 438 to 506), which is connected to two adjacent β-strand of the core; the RBM becomes the most membrane distal site of the S in its receptor-binding open conformation (8, 9). The RBD is the main antigenic site in CoVs and the target of potent neutralizing Abs (12, 13). In SARS-CoV-2, Abs bind to different RBD regions or subsites, either at the core or RBM (3, 14, 15). Based on their RBD epitopes, Abs have been classified in at least 4 different classes. Some are RBD conformation-specific, while others can recognize its open and closed forms.

Since the begining of the pandemic, SARS-CoV-2 circulating strains acquired mutations that facilitate its transmission, like the S2 D614G substitution that replaced the original Wuhan (WA1) strain to become dominant globally (16). Current SARS-CoV-2 variants contain additional mutations in the RBD (17) that enhance virus transmission by increasing affinity for human ACE2 (e.g. N501Y, K417N/T, L452R) and facilitate escape from neutralizing antibodies (Abs) (e.g. E484K, L452R) (18–22). Among current variants, the B.1.1.7 (alpha), first detected in the United Kingdom, and the B.1.1.28 or P.2 (zeta) initially identified in Brazil, carry the single RBD mutations N501Y and the E484K, respectively. Nonetheless, other variants of concern contain multiple RBD mutations, as the B.1.351 (beta), reported initially at South Africa with the substitutions K417N, E484K and N501Y, or the related B.1.1.28.1 or P.1 (gamma), which contains changes at the same residues, but with K417T. We have also attended to the global dissemintation of highly contagious virus variants that rapidly have become prevalent. The B.1.617.2 (delta) variant, and its related B.1.617.1 (kappa), were identified initially in India and carry RBD L452R-T478K or the L452R-E484Q substitutions, respectively, which are likely responsible of their enhanced transmission. More recently, the B.1.1.529 (omicron) variant, identified in South Africa, contains an extreme evolution of the RBD with fifteen substitutions (i.e. G339D, S371L, S373P, S375F, K417N, N440K, G446S, S477N, T478K, E484A, Q493K, G496S, Q498R, N501Y, Y505H) (23). The RBD mutations found in these variants pose a threat to the efficacy of current vaccines and human therapeutic Abs (22, 24, 25), which may also generate an additional pressure for viral evolution and selection of new variants that escape human immune neutralization. In this context, it becomes highly important the identification of neutralizing Abs with therapeutic potential and binding epitopes distinct from those recognized by Abs in humans.

Heavy chain Abs (hcAb) derived from single variable V_HH_ domains or Nanobodies (Nbs) naturally found in camelids (e.g., dromedaries, llamas, alpacas), hold a great potential given their unique structural, biophysical, and epitope-binding properties (26). Despite their small size (~14 kDa), Nbs show identical affinity and antigen specificity as conventional Ab molecules with heavy and light chains (i.e., human/mouse IgGs); they have superior properties such as enhanced stability to thermal and chemical denaturation, higher solubility, resistance to proteolysis, and the ability to bind to small protein cavities and conserved inner regions on pathogen surfaces, which are hidden to conventional Abs (e.g. IgGs) to avoid immune neutralization (27, 28). Nbs can be expressed as monomeric, monovalent molecules, or as fusions with other Nbs and/or protein domains (e.g. Fc of human IgGs) producing bivalent and multivalent molecules with mono-, bi-, or multi-specific binding capabilities (29). These properties provide important advantages in the use of Nbs for diagnostic and therapeutic applications (30). In addition, Nbs share high sequence identity with human VHs and can be readily humanized or directly applied in therapy (31). Nbs (or fusions containing them) can be delivered intravenously as conventional Abs and by direct inhalation in the lung (32).

Nbs have been developed against different viral infections (33). Since the begining of the COVID-19 pandemic, different V_HH_ libraries have been screened to isolate Nbs that bind the RBD of SARS-CoV-2. Various Nbs neutralizing viral SARS-CoV-2 WA1 strain *in vitro* were reported (34–41). More recent studies also reported Nb binding to the RBD of alpha, beta, and gamma variants and that neutralized SARS-CoV-2 *in vitro* (42, 43). Few studies have demonstrated *in vivo* protection of SARS-CoV-2 infection with Nbs, or characterized their binding to circulating variants (44, 45).

Here, we report a panel of high affinity Nbs binding to diverse SARS-CoV-2 RBD epitopes, and a set of Nb-derived neutralizing hcAbs with therapeutic potential *in vivo*, as they can protect hACE2-transgenic mice after infection with a lethal dose of SARS-CoV-2. A broad Nb/hcAb characterization using biophysical, cell and structural biology methods showed how they recognized distinct RBD sites with very high affinities; most of them blocked RBD binding to its ACE2 receptor and inhibited SARS-CoV-2 cell entry. In addition, we determined the specificity of the neutralizing hcAbs for major RBD variants (alpha, beta, gamma, kappa, zeta, delta and omicron), and identified mono- and bi-specific hcAbs that recognized with high affinity all variant RBDs except omicron. Our data showed the therapeutic potential of these Nbs and hcAbs against most SARS-CoV-2 variants and highlighted the antibody escape capacity of omicron.

## Materials and Methods

### Dromedary Immunization and VHH Library Construction

Two adult and healthy dromedary camels (*Camelus dromedarius*) were immunized by subcutaneous injection of purified RBDmFc protein (described below) once in every week during six weeks. Every injection contained 0.2 mg of RBDmFc in 2 ml sterile saline (0.9% NaCl) and 2 ml of adjuvant (FAMA Adjuvant, GERBU). The total volume (4 ml) was injected in three distinct sites in the camel. Serum samples were collected before the first immunization and four days after the last boost, and they were used to confirm Ab immune response by ELISA. An additional 50 ml of blood was collected from the jugular vein in tubes containing EDTA as anticoagulant (K2EDTA Tubes, Vacutainer^®^, BD) for lymphocyte isolation. Lymphocyte mRNA extraction, first-strand cDNA synthesis and PCR amplication of VHHs was conducted as previously reported (46) with oligonucleotide primers VHHSfi2 (5’-GTC CTC GCA ACT GCG GCC CAG CCG GCC ATG GCT CAG GTG CAG CTG GTG GA) and VHHNot2 (5’-GGA CTA GTG CGG CCG CTG AGG AGA CGG TGA CCT GGGT). The amplified VHH fragments from dromedary 1 (D1) and dromedary 2 (D2) were digested with *Sfi*I and *Not*I restriction enzymes, ligated into the same sites of pNeae2 vector and electroporated separately into *E. coli* DH10B-T1R cells for surface display of the Nbs as fusions with the intimin anchoring domain (47, 48).

### Nanobody Surface Display in Bacteria

The bacteria that displayed the D1 and D2 Nb libraries were grown at 30 °C in Lysogeny broth (LB) liquid medium, or on LB-agar 1.5% (w/v) plates, both with chloramphenicol (Cm, 30 μg/ml) and 2% (w/v) glucose (Glu) for repression of the *lac* promoter. For induction of Nb fusions, bacteria were grown on liquid LB-Cm (without Glu) containing 0.05 mM isopropylthio-β-D-galactoside (IPTG). Details of growth and induction conditions are reported in the **Supplementary Materials and Methods**.

### Selection of RBD-Binding Nbs From Immune Libraries Displayed on *E. coli*

Duplicated bacterial samples representing each library (D1 and D2) with ~5x10^8^ CFU in 100 μl of PBS-BSA (phosphate buffered saline with 0.5% w/v of bovine serum albumin) were mixed in a total volume of 200 μl with biotinylated RBD in the same buffer at 100 nM or 50 nM for the first and second magnetic cell sorting (MACS) steps, respectively. After 1 h incubation at RT, bacteria were washed, incubated with anti-biotin paramagnetic beads (Miltenyi Biotec), loaded onto a MACS MS column (Miltenyi Biotec), and bound bacteria recovered as described in the **Supplementary Materials and methods**. After a second MACS cycle, the bound bacteria were selected by fluorescence activated cell sorting (FACS). The induced bacteria (~5x10^8^ CFU) were mixed in a total volume of 200 μl of PBS-BSA with biotinylated RBD (50 nM final concentration) and mouse anti-c-myc monoclonal Ab (1:500, 9B11 clone, Cell Signaling), which labels intimin-Nb-myc tag fusions expressed on the bacterial surface. After 1 h incubation at RT, bacteria were washed and stained with goat anti-mouse IgG-Alexa Fluor 488 conjugated polyclonal Ab (1:500; ThermoFisher Scientific) and Streptavidin-Allophycocyanin (APC) (1:100; SouthernBiotech). After 1 h incubation at 4°C (in the dark), bacteria were washed and resuspended in PBS for sorting in a FACS vintage SE cytometer (Becton Dickinson). At least ~1x10^6^ bacterial events were processed per sample. Double-stained bacterial population with high fluorescence intensity signals was collected (at least ~5x10^3^ events) in LB-Cm-Glu medium and plated (see **Supplementary Materials and Methods** for further details).

### Flow Cytometry Analysis of Antigen Binding to *E. coli* Bacteria Displaying Nbs

Binding of biotin-labeled antigens (**Supplementary Materials and Methods**) to Nbs displayed on bacteria from libraries (enriched pools or individual clones) were analyzed by flow cytometry. To this end, induced bacteria cultures were washed and incubated with the indicated biotin-labeled antigen (i.e., 5 nM RBD, 5 nM S, or 100 nM human fibrinogen) and anti-c-myc monoclonal Ab, as described above for FACS selection. Bacteria were stained with secondary fluorophore reagents as for FACS selection and analyzed in a Gallios cytometer (Beckman Coulter).

### Expression and Purification of hcAbs and Nbs

The DNA segments coding for the selected Nbs were PCR amplified from bacterial pNeae2-VHH plasmids with oligonucleotide primers Nb1-N2-pIg-AgeI (5’-ACT GCA ACC GGT GTA CAT TCT CAG GTG CAG CTG GTG GAA) and Nb1-C5-pIg-BamHI (5’-ACT GGA TCC AGA ACC ACT TGC CGC TGA GGA GAC GGT GAC CTG) and cloned in *Age*I-*Ba*mHI sites in frame with a IgH signal peptide and the human IgG1 hinge and Fc portion (Fc) of mammalian expression vector pIgΔCH1, derived from pIgγ1HC (kindly provided by Prof. M. Nussenzweig, Rockefeller University) (49). The hcAbs were routinely produced in mammalina cells with these recombinant plasmids. For Nb preparation, we generated constructs with a thrombin recognition site (LVPRGS) between the Nb domain and the Fc using oligonucleotide primers Nb1-N2-pIg-AgeI (see above) and Nb1-C2-pIg-BamHI (5’-TCA CTC GAG GCG GAT CCA CGC GGA ACC AGC GCT GAG GAG ACG GTG AC) and cloned between *Age*I-*Ba*mHI sites of pIgΔCH1. In the bispecific hcAbs, a GS-linker enconding (GGGGS)x3 sequence was cloned between the C-terminus of the first VHH domain and the N-terminus of the second VHH domain. To this end, the second VHH domain was amplified with the GS-linker using oligonucleotide primers BamHI-Nb-N-linker (5’-ATG GGA TCC GGA GGT GGC GGG TCC GGA GGT GGC GGG TCC GGA GGT GGC GGG TCC CAG GTG CAG CTG GTG GAG TCT) and Nb1-C5-pIg-BamHI (see above) and cloned in the *Bam*HI site of the pIgΔCH1 vector carrying VHH1.

In all cases, suspension human embryonic kidney (HEK-293F) mammalian cells were transfected with purified expression vector DNAs using polyethylenimine (PEI) and following standard procedures. Cultures with the proteins in supernatants were collected 5 days post-transfection. Cells were then centrifuged, and proteins purified from the cell supernatant with an Ig Select or protein A affinity columns (Cytiva), as indicated by the manufacturer. The Ab samples were further purified by size exclusión chromatography (SEC) with a Superdex75 (10/300) column (Cytiva) in HBS (20 mM HEPES, 150 mM NaCl, pH 7.4). Single Nbs were generated by thrombin digestion of hcAb proteins with a thrombin site after the VHH domains (see above). The Fc and Nb portions were separated by SEC with a Superdex75 (16/600) column (Cytiva) in HBS.

### Production of the S and RBD Proteins

A recombinant DNA fragment coding for the soluble S (residues 1 to 1208) protein was obtained by gene synthesis (GeneArt, ThermoFisherScientific) and cloned betweem *Xho*I-*Not*I sites of pcDNA3.1 vector for expression in HEK-293F cells (50). The recombiant DNA construct contained the S signal sequence at the N-terminus, and a T4 fibritin trimerization sequence, a Flag peptide and an 6x or 8xHis-tag at the C-terminus. In the S protein, the furin-recognition motif (RRAR) was replaced by the GSAS sequence, and it contained the A942P, K986P and V987P substitutions in the S2 portion (51). The soluble S was purified by Ni-NTA affinity chromatography from transfected suspension HEK293F cell supernatants, and it was transferred to HBS pH 7.5, during concentration or by SEC in a Superose 6 (10/300) column.

Several RBD constructs have been used in this study. In some constructs, synthetic DNA fragments encoding for the RBD and a thrombin site (GeneArt, ThermoFisherScientific) were cloned between the *Age*I and *Bam*HI sites of pIgΔCH1 (see above); in others, the PCR-amplified RBD portion was cloned in frame with the IgK leader sequence and an HA-tag (YPYDVPDYA), and contained thrombin recognition sites (LVPRGS) at the 5’ and 3’ RBD ends. Initially, we also prepared a recombinant RBD (residues 334–528 of the S) C-terminal fused to the TIM-1 mucin domain and the human IgG1 Fc region (RBDmFc) in HEK293F cells (52), which was used for camel immunization. Subsequently, we produced RBD-Fc fusion proteins without the mucin domain and with S residues 332 to 534, as well as a monomeric or trimeric RBDs (332–534) without or with a T4 fibritin trimerization sequence, respectively, and with a Flag peptide and an 8xHis-tag at the C-terminus (RBD-FH or RBD-TFH). The recombinant RBD constructs in mammalian expression vectors were used for protein production by transient transfection of HEK293F cells. The RBD-Fc proteins were purified as described for hcAbs, whereas the His-tagged RBDs were prepared by Ni-NTA affinity chromatography.

### Enzyme-Linked Immunosorbent Assays (ELISA) to Evaluate hcAb Binding to S and RBD Proteins

Different assays were conducted to test binding of camel sera or purified hcAbs to RBD and S proteins. 96-well ELISA plates (Maxisorp, Nunc) were coated o/n at 4 °C with 50 µl/well of the indicated protein antigen (RBD-TFH, RBD-Fc or S), or BSA as negative control, at ~3 µg/ml in PBS, or 10 mM Tris-HCl 50 mM NaCl, pH 7.4. Specific conditions of blocking, incubation with primary (camel sera or hcAbs) and secondary reagents conjugated to horseradish peroxidase (HRP), and development are described in the **Supplementary Materials and methods**. Optical Density at 490 nm (OD_490_) values were corrected with the background OD_490_ of wells without antigen or with BSA.

### hcAb Competition of RBD-hACE2 Interaction

ELISA plates (Maxisorp, Nunc) were coated o/n at 4^°^C with 5 µg/ml of the soluble extracellular domain of hACE2 (residues 19-615) in PBS (50 µl/well). Soluble hACE2 with a C-terminal His-tag was purified from insect cells (a kind gift of Dr. José F. Rodríguez, CNB-CSIC). After coating, plates were washed with PBS and blocked at RT for 2 h with 200 μl/well of 3% (w/v) skimmed milk and 1% (w/v) BSA in PBS. A dilution of the biotin-labeled RBD-Fc at 20 nM was prepared in 1% (w/v) skimmed milk-PBS containing the indicated concentrations of unlabeled hcAb competitor (i.e. 0, 50, 100, or 200 nM), and the mixture was added to hACE2-coated immunoplates for 90 min at RT. After incubation, the wells were washed five times with PBS and the binding of biotinylated RBD-Fc detected with secondary streptavidin-HRP (1:1000; Merck-Sigma) in 1% (w/v) skimmed milk-PBS. After 45 min incubation at RT, plates were washed, developed, and OD_490_ determined as above.

### Binding Kinetics and Affinity Determination by Surface Plasmon Resonance (SPR)

SPR was applied to monitor hcAb/Nb binding to S/RBD proteins in a BIAcore 3000 instrument (GE Healthcare) with CM5 chips in HBS buffer. An anti-Flag Ab (M2, Merck-Sigma) was covalently immobilized into the dextran surface of the chip with the “amine coupling kit” (BIAcore) to capture S or RBD-FH proteins. In each cycle, 15 µl (20-30 µg/ml) of S or RBD-FH were injected at 5 µl/min, followed by the injection of 40 µl of hcAb or Nb at 10 µl/min in HBS. Chip surfaces were regenerated with a 5 µl pulse of 50 mM phosphoric acid at 100 µl/min. Two surfaces (Fc2 and Fc3) with immobilized anti-Flag Ab (5000 and 8500 RU) were monitored in each experiment. Binding kinetics were determined by the analysis of the sensorgrams with the BIAEvaluation 3.0 software after the subtraction of the signal from an empty flow cell surface (Fc1); no meaningful (1.7 ± 1 RU) unspecific Nb/hcAb binding to an anti-Flag Ab surface without captured ligands (S/RBD) was recorded after Fc1 correction. In addition, baseline drifting due to ligand dissociation from the Flag Ab was corrected with a sensorgram recorded with HBS injection (double referencing). The sensorgrams were adjusted to a Langmuir (1:1) model for binding kinetics estimation, which were determined following separate and simultaneous fitting procedures in the BIAevaluation program, and then, averaged.

### hcAb Neutralization of Pseudotyped Retroviral Particles *In Vitro*

The neutralization capacity of hcAbs was first determined using luciferase-based reporter retroviral particles pseudotyped with the S protein of the SARS-CoV-2 WA1 strain (Spp). Pseudotyped viruses were produced by transfection of the S protein expressing plasmid together with packaging plasmids in HEK-293T cells as previously described (50). Retroviral particles pseudotyped with G glycoprotein (VSVGpp) from the vesicular stomatitis virus (VSV) were produced and used in parallel as control. Luciferase reporter activity was used to titrate the pseudotyped viruses in Vero-E6 cells and with complete DMEM + 2% Fetal Bovine Serum (FBS).

The hcAbs were diluted in 50 μl of complete DMEM + 2% FBS at 4, 0.4, and 0.04 μg/ml, mixed with 50 μl of pseudotyped virus and kept for 1 h at 37°C. The mixtures were added to wells of flat-bottom 96-well plates with the Vero-E6 (1x10^4^ cell/well), and incubated ~20 h at 37°C, after which the medium was replaced by 100 μl/well of complete DMEM + 2% FBS. Plates were further incubated for additional 24 h at 37°C. The next day (48 h.p.i.) medium was discarded and cell lysates were prepared in passive lysis buffer (Promega). Luciferase activity assay was performed as indicated by the manufacturer (Promega). Activity in wells without hcAb were used as reference and set to 100%.

### hcAb Neutralization of SARS-CoV-2 *In Vitro*

Neutralization of SARS-CoV-2 virus (strain NL/2020) was carried out in Vero-E6 cells at the CNB-CSIC BSL3 facility. This viral strain was isolated from an infected patient in the Netherlands in February 2020 and was obtained through the EVAg repository from Dr. Molenkamp (Erasmus University-Rotterdam,NL). As described for pseudovirus neutralization experiments, hcAb-virus mixtures (100 μl/sample) with 140 FFU (MOI of 0.007) of SARS-CoV-2 were added to wells of flat-bottom 96-well plates with Vero-E6 (2x10^4^ cell/well), and incubated for 1 h at 37°C. After the incubation, the infective mixtures were removed, the cells were washed once with complete PBS and 100 μl/well of complete DMEM+2% FBS added. Plates were incubated for 24 h at 37°C, before medium was discarded and cells fixed with a 4% formaldehyde-PBS solution for 20 min at RT, after which fixative was discarded and cells were extensively washed with PBS. Cells were then incubated in binding buffer (3% BSA; 0.3% Triton X100 in PBS) for 1h at RT, and immunofluorescence performed using a primary rabbit monoclonal antibody against N protein (Genetex) diluted 1:2000 in binding buffer. After 1h incubation at RT, cells were washed with PBS and subsequently incubated with a 1:500 dilution of a secondary goat anti-rabbit conjugated to Alexa 488 (Invitrogen) antibody. Cells were counterstained with Dapi (Life Technologies) during the secondary antibody incubation time. Automated fluorescence microscopy imaging was performed in a SparkCyto (Tecan) multimode plate reader. Immunofluorescence signal in wells without hcAb were used as reference and set to 100%.

### hcAb Inhibition of COVID-19 Progression in Mice

A total of 36 six-week-old hemizygous K18-hACE2 transgenic (B6.Cg-Tg(K18-ACE2)2Prlmn/J) female mice were used (The Jackson Laboratory, ME, USA). Animals were anesthetized under isoflurane and inoculated intranasally (i.n.) with 50 µl of DMEM containing 5×10^4^ PFU of SARS-CoV-2 isolate hCoV-19/Spain/SP-VHIR.02, D614G(S) from lineage B.1.610, kindly provided by Dr. Miguel Chillón (Universitat Pompeu i Fabra, Barcelona, Spain). Uninfected control mice were treated in parallel with DMEM. Six mice per group were inoculated intraperitoneally (i.p.) with 150 µg of each hcAb (equivalent to ~8 mg/kg for a mouse of ~18 g) in 150 µl of HBS at 24 h.p.i. Uninfected control mice were inoculated at the same time only with buffer. Animals received water and food *ad libitum* and were monitored daily for clinical signs and body weight. Euthanasia was applied when the animals exhibited irreversible disease signs. All surviving mice were anesthetized and euthanized at the end of the experiment.

### Cryo-EM of the S-Nb Complexes

Ni-NTA affinity purified S with 6xHis-tag was mixed with the Nb (1:1.5 molar ratio of S monomer:Nb) during 30 min at 10°C, and the S-Nb complex purified by size exclusion chromatography in a Superose 6 (16/60) column with 20 mM Tris-buffer and 200 mM NaCl, pH 7.7. The sample was concentrated to 1mg/ml and applied to glow-discharged holey carbon grids (Quantifoil, Au 300 mesh, R 0.6/1). The grids were blotted and then plunged into liquid ethane using a FEI Vitrobot Mark IV at 4°C. Data were collected at a FEI Talos Arctica electron microscope operated at 200 kV and equipped with a Falcon III electron detector; a dataset was collected in a Titan Krios instrument at the ESRF CM01 line. At the Talos Arctica, the images were recorded at a defocus range of -1 μm to -2.5 μm with a pixel size of 0.855 Å; exposure time was 40 s, with a total exposure dose of 32 e/Å^2^ over 60 frames. At the Titan Krios, a super resolution acquisition mode was used with a 105k magnification and a pixel size of 0.84 Å, with a defocus range of -1.0 to -2.4 um in 0.2 um step; the exposure time was of 1.8 s, with a total exposure dose of 39.0 e/Å^2^ (15.3 e/pixel/s) over 40 frames.

Particle reconstructions were carried out throughout INSTRUCT projects in the Electron Microscopy Image Processing, I2PC, Madrid, which resulted in EM maps of the trimeric S with the bound Nbs. We used Scipion 2.0 (53) in order to easily combine different software suites in the analysis workflows of CryoEM data: Movie frames were aligned using MotionCor2; the contrast transfer function (CTF) of the micrographs was estimated using CTFFIND4; particles were automatically selected from the micrographs using autopicking from Gautomatch. Evaluation of the quality of particles and selection after 2D classifications, the initial volume for 3D image processing, the 3D-classification and the final refinement were calculated using cryoSparc, whereas the sharpening was estimated by DeepEMhancer.

Model building for the S-Nb complexes was performed with a S protein structure (PDB 6ZXN) and Nb models, which were prepared with the program Modeller based on the 1ZV5 (1.10), 3TPK (1.29) and 6DBA (2.15) structures. The S structure and the Nb models were fitted into the EM map with the chimera and coot programs. Subsequently, the structures were subjected to real-space refinement in PHENIX (54), which included cycles of rigid body, global and adp minimization. NCS was applied among the S domains, but excluding the RBDs. Nb-RBD binding interfaces were determined with the PISA server, and main figures of the structures were prepared with pymol (pymol.org). The EM maps and the refined structures have been deposited in the PDB with accession codes 7R4R, 7R4Q and 7R4I for the S-1.10, S-1.29 and S-2.15, respectively.

### Statistics

Means and standard errors (SEM), or standard deviations (SD), of experimental values from independent experiments (*n*, as indicated in Figure Legends) were calculated using Prism 7.0 and 8.0 (GraphPad software Inc.). Binding curves represented using GraphPad Prism performing a non-linear regression (curve fit). Differences in binding were evaluated using two-tailed paired t-test. Log-rank (Mantel-Cox) test was used for survival analysis of mice using Prism 7.0 (GraphPad software Inc).

## Results

### Selection of a Panel of Nbs Binding to the RBD of SARS-CoV-2

**Figure 1:**
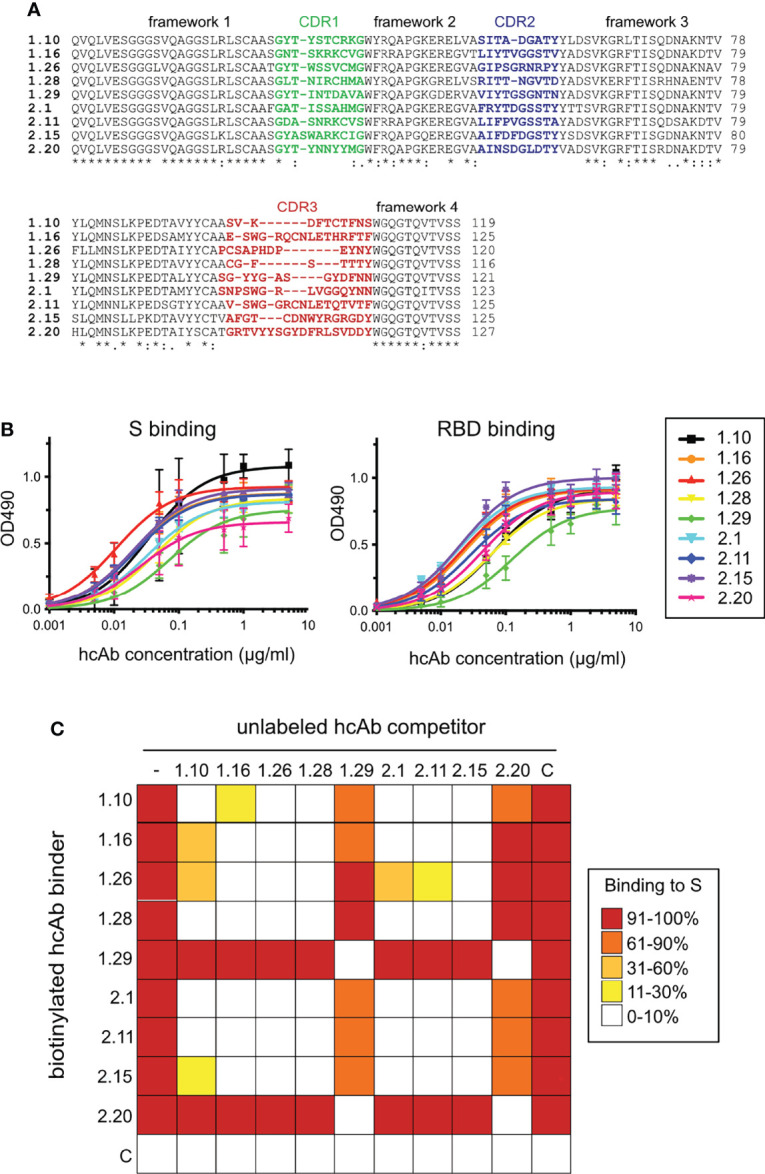
SARS-CoV-2 RBD-specific Nbs, binding to S and RBD proteins, and cross-competition analysis. **(A)** Sequence alignment of the 1.10, 1.16, 1.26, 1.28, 2.1, 2.11, 2.15 and 2.20 Nbs selected from bacterial libraries that displayed VHH domains isolated from two immunized dromedaries with the SARS-CoV-2 RBD (**Figure S1**). Their VHH frameworks and complementarity determining regions (CDRs) 1 to 3 are indicated. Alignment generated with Clustal Omega (55). Labels indicate full conservation (*) or degree of conservation (: or .). **(B)** Binding of Nb-derived hcAbs to the S (left) or RBD (right). The hcAbs contained the RBD-specific Nb domains fused to the human IgG1 hinge and Fc portion (**Figure S2A**). Binding of serial hcAb dilutions to plastic-bound proteins measured as Optical Density at 490 nm (OD_490_) as described in *Materials and methods*. Average and standard deviations (n ≥ 3). **(C)** S binding competition among hcAbs. Heatmap representation of the binding data shown in **Figure S3**, and determined with biotin labeled hcAbs (left) without (-) or with the unlabeled hcAb competitor shown on the top. A control hcAb (C) was also included. Mean of three independent experiments (n = 3).

We immunized two dromedary camels using the RBD of the SARS-CoV-2 from the WA1 strain fused to a mucin domain and the Fc portion of human IgG1 (RBDmFc in **Figure S1A**; Materials and methods). ELISA of sera from both immunized animals showed specific Ab response against the RBD alone (**Figure S1B**). A blood sample of each immunized dromedary was used for RT-PCR amplification of the VHHs from lymphocyte mRNA samples (Materials and methods). Each VHH pool was cloned into the pNeae2 vector for Nb display on the *E. coli* bacterial surface (47, 48), generating two immune libraries of VHHs (D1 and D2) with ~6.2x10^7^ and ~3.5x10^7^ independent clones, respectively. Bacterial clones binding to monomeric RBD (**Figure S1A**; Materials and methods) were enriched from each library by two cycles of magnetic cell sorting (MACS), followed by one cycle of fluorescence-activated cell sorting (FACS) (**Figure S1C**). From the FACS, approximately 5x10^3^ events with positive fluorescence signal were collected from each library and plated. Sixty individual colonies (30 from each library) were randomly picked, grown and analyzed by flow cytometry for binding to monomeric RBD and trimeric S proteins. Twelve clones showed strong binding signals at low concentrations (5 nM) of RBD or S proteins, and no detectable binding to 100 nM of human fribrinogen (control antigen). DNA sequencing of the selected VHHs clones allowed us to identify 5 distinct Nb sequences from the D1-pool (1.10, 1.16, 1.26, 1.28, 1.29) and 4 Nb sequences from the D2-pool (2.1, 2.11, 2.15 and 2.20). Nb 1.10 was found in two independent colonies and Nb 1.26 in three clones. The other Nbs were unique among the analyzed colonies. Flow cytometry demonstrated specific binding of the RBD and S proteins to bacteria displaying these Nbs (**Figure S1D**). Alignment of the amino acid sequence of the selected Nb clones binding the RBD of SARS-CoV-2 is shown in **Figure 1A**, indicating their unique CDRs.

### Production of Nbs and hcAbs, Ligand Binding and Cross-Competition

We constructed hcAbs by the fusion of the selected VHH domains to the Fc region of human IgG1 (Fc) in a mammalian expression vector (**Figure S2A**, Materials and methods). Nine hcAbs were generated from the corresponding VHH clones and purified from culture supernatants of transfected 293F cells (**Figure S2B**, top). Monomeric Nbs were prepared after thrombin digestion of hcAbs with an engineered thrombin cleavage site between the Nb and Fc moieties (**Figure S2B**, bottom).

Initially, binding of hcAbs to the S and RBD ligands was analyzed by ELISA, using serial hcAb dilutions and immunoplates coated with the SARS-CoV-2 antigens. The nine hcAbs showed similar binding curves to trimeric S or RBD proteins (**Figure 1B**). The half-maximal effective concentration (EC_50_) for hcAb binding to the S ranged from 0.01 μg/ml (0.25 nM) for 1.26 to 0.07μg/ml (1.8 nM) for 1.29, whereas EC_50_ for binding to RBD ranged from 0.02 μg/ml (0.5 nM) for 2.15 to 0.12 μg/ml (3 nM) for 1.29. These data confirmed the S and RBD binding potency of the Nbs selected by bacterial display.

To gain insights on the hcAb epitopes in the SARS-CoV-2 RBD, we carried out cross-binding competition assays. Binding of a biotin-labeled hcAb (0.1 μg/ml, 2.5 nM) to plastic-bound S was determined by ELISA without or with 200-fold molar excess of unlabeled hcAbs (**Figure S3**), and it is summarized as a heat map in **Figure 1C**. These experiments identified two distinct binding epitopes or Nb classes in the panel. Most hcAbs (1.10, 1.16, 1.26, 1.28, 2.1, 2.11, 2.15) cross-competed for S binding and they were clustered in the same A group, whereas binding of the 1.29 and 2.20 hcAbs to S, which cross-competed, was not inhibited by the others, and thus formed a distinct B group. Competition data also suggested certain epitope variability among the A group Nbs. For instance, hcAbs 1.10 or 2.1 did not blocked completely 1.26 or 2.15 binding to S (**Figure 1C** and **Figure S3**). Together, these data suggested that the Nbs preferentially targeted two distinct non-overlapping RBD regions, whereas group A Nbs had overlapping but still specific epitopes, determined by their unique CDRs.

### Affinity and Kinetic Constants for Nb and hcAb Binding to S and RBD Ligands

**Figure 2:**
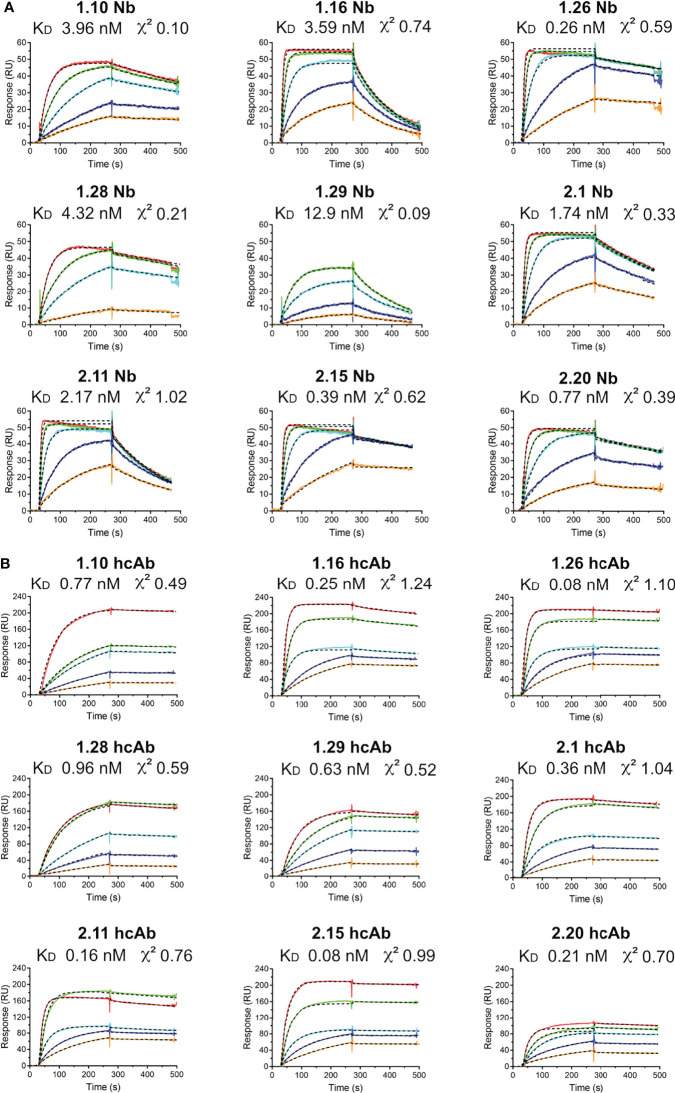
Nb and hcAb binding to the SARS-CoV-2 RBD ligand in real time. Overlayed sensorgrams recorded during the association and dissociation of the indicated Nbs **(A)** or hcAbs **(B)** through BIAcore sensor chip surfaces with captured RBD. Nb or hcAb concentrations of 5 (orange), 10 (blue), 25 (cyan), 50 (green) or 100 nM (red) were injected through the RBD and a control surface. The plots show the specific response (RU) after double referencing (see *Materials and methods*). The discontinuous dark lines represent the curve fitting to a 1:1 Langmuir model for determination of the kinetic constants, shown in **Table S1**. The equilibrium dissociation contents (K_D_) and the fitting χ^2^ are indicated here.

Surface plasmon resonance (SPR) was used to establish the kinetic constants for hcAb and single domain Nb binding to the RBD or S ligands. To achieve homogenous surfaces and more accessible epitopes, protein ligands were produced with an C-terminal Flag-tag and captured with an anti-Flag monoclonal Ab (mAb) covalently bound to a dextran sensor chip (CM5, BIAcore). After its capture, Nbs or hcAbs were injected through surfaces with and without ligand (RBD or S), which allowed the determination of specific binding (**Figures 2**, **S4** and **Materials and Methods)**. As expected for their higher mass and dimeric nature, resonance units (RUs) were 3-4 times higher with the hcAbs compared to the Nbs (**Figures 2**, **S4**). The corrected sensorgrams were analyzed for determination of the association (*k_ass_*) and dissociation (*k_dis_*) kinetic rate constants (**Table S1**), which were used to calculate of the equilibrium dissociation constants (K_D_) for each Nb/hcAb binding to the RBD or S, shown in **Figures 2**, **S4**, respectively. The monomeric Nbs showed consistent low and sub-nanomolar affinities in binding to RBD and S, with the K_D_ ranging from 13 nM for 1.29 Nb binding to RBD to ∽0.3 nM for 1.26 or 2.15 binding to either RBD or S. Nb dimerization in the hcAbs reduced at least 10-fold their *k_dis_* from the ligand (**Table S1**), whereas the *k_ass_* did not change for RBD binding or reduced slightly (~2-fold) for S binding. Indeed, some Nbs bound faster to the S than their corresponding hcAbs (**Figure S4**), likely due to its smaller size. Also, the similar Nb association rates to RBD alone and to the S indicated a good accessibility of their binding sites in the S trimer. Notably, the 1.26 and 2.15 hcAbs bound to S and RBD with affinity values in the picomolar (pM) range and all the hcAbs showed low *k_dis_* (~10^-4^ s^-1^; **Table S1**).

### hcAb Inhibition of SARS-CoV-2 RBD Binding to Its Cell Entry Receptor

**Figure 3:**
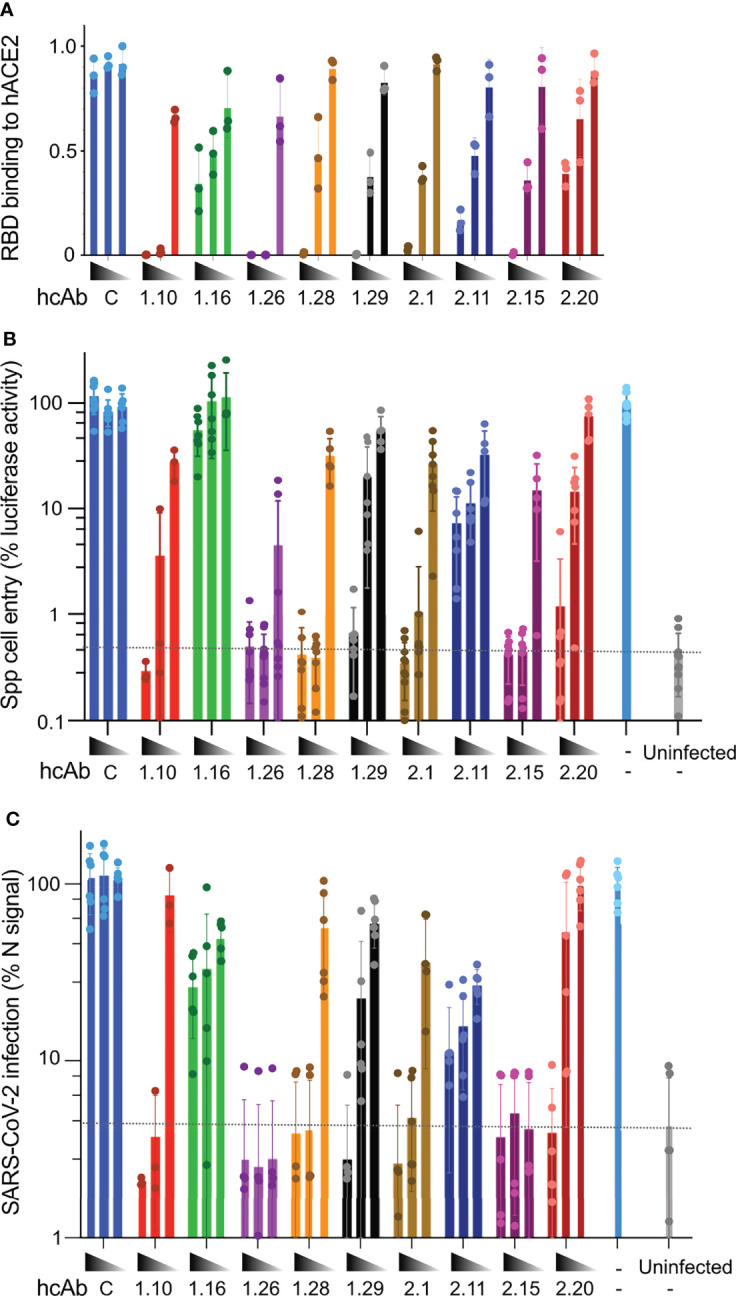
hcAb inhibition of RBD binding to ACE2 and SARS-CoV-2 cell infection *in vitro*. **(A)** RBD binding to hACE2 in the presence of the indicated hcAbs (1.10, 1.16, 1.26, 1.28, 2.1, 2.11, 2.15, 2.20) or a control hcAb (C). Immunoassays with biotinylated RBD-Fc (20 nM) and increasing hcAb concentrations (50, 100 and 200 nM, from right to left). Mean and SD from three independent assays (n = 3). **(B)** Inhibition of Vero-E6 cell entry of pseudotyped viral particles with SARS-CoV-2 S protein (Spp) by the indicated hcAbs at increasing concentrations (0.5, 5 and 50 nM). The luciferase activity of cell cultures was determined 48 h.p.i. Background luciferase activity of uninfected cell cultures is shown with a dashed line. **(C)** Inhibition of SARS-CoV-2 virus cell infection by the indicated hcAbs as in **(B)** Infection efficiency was determined by immunofluorescence 24 h.p.i. by staining with anti-N monoclonal Ab (*Materials and methods*). Background fluorescence detected in uninfected cell cultures is shown with a dashed line. **(B, C)** Infected cell cultures without hcAb (-) used as positive controls for Spp and SARS-CoV-2 infections.

SARS-CoV-2 attaches to its cell surface ACE2 receptor through the RBD, which initiates the virus cell entry process (7). To further characterize the RBD-specific Nbs, we evaluated their ability to compete RBD binding to human ACE2 (hACE2). To this end, we compared the binding of biotin-labeled RBD-Fc (20 nM) to hACE2-coated immunoplates without or with an excess of each hcAb at increasing concentrations (from 50 to 200 nM) (**Figure 3A**). Except for 1.16, 2.11 and 2.20, the selected hcAbs fully inhibited (≥95%) RBD binding to hACE2 at 10-fold molar excess (200 nM). At this concentration, hcAbs 1.16, 2.11 and 2.20 only reduced RBD binding to 20-40% of the maximal signal, whereas a control (C) hcAb had no significant effect (**Figure 3A**). Notably, 1.10 and 1.26 fully inhibited RBD-hACE2 interaction at 5-fold molar excess (100 nM), showing that they were the most potent inhibitors of the SARS-CoV-2 RBD-receptor interaction. Other group A (2.1 and 2.15) and one group B (1.29) hcAb were able to inhibit RBD binding over 50% at 100 nM, whereas the remaining hcAbs showed poorer inhibition activity at this concentration.

### hcAb Neutralization of S-Pseudotyped and SARS-CoV-2 Viruses *In Vitro*

The ability of the hcAbs to inhibit RBD binding to hACE2 should contribute to SARS-CoV-2 neutralization, as it would prevent virus cell entry. We then tested the hcAb neutralization of luciferase-enconding retroviruses pseudotyped with the WA1 strain SARS-CoV-2 S (namely, Spp), or with the G glycoprotein of Vesicular Stomatitis virus (VSVGpp) as a negative control. Vero-E6 cells were cultured with Spp or VSVGpp preincubated without and with the hcAbs at increasing concentrations (0.5, 5, and 50 nM), and infectivity measured as cell associated luciferase at 48 hours post infection (h.p.i.). All but the 1.16 and the 2.11 hcAbs inhibited Spp cell entry with diverse efficacy (**Figure 3B**). The 1.26 hcAb, which bound to S with picomolar affinity (**Table S1**), was the most effective and prevented at least 90% of the cell infection at any of the tested concentrations. Similarly, other group A hcAbs such as 1.10, 1.28, 2.1 and 2.15, which blocked RBD binding to hACE2, inhibited more than 90% the infection at 5 and 50 nM (**Figure 3B**). The 1.29 and the 2.20 group B hcAbs showed at least 90% neutralization at 50 nM (**Figure 3B**). No inhibition of Spp cell entry was observed with a specificity control (C) hcAb at any of the concentrations tested (**Figure 3B**). Also, no inhibition of VSVGpp cell entry was detected with any of the hcAbs tested (**Figure S5**).

Next, we performed *in vitro* neutralization assays with the SARS-CoV-2 virus, and the same hcAb concentrations used above (0.5, 5, and 50 nM). In this assay, viruses were preincubated with the hcAbs and added to Vero-E6 cells for 1 h before they were removed by washing, and cell infection was quantified by immunofluorescence microscopy at 24 h.p.i. These experiments (**Figure 3C**) showed complete inhibition (>90%) with the two highest concentrations tested of the group A hcAb that fully inhibited RBD binding to hACE2 (1.10, 1.26, 1.28, 2.1, and 2.15). Notably, 1.26 and 2.15 showed complete SARS-CoV-2 neutralization even at 0.5 nM, indicating a potent neutralization activity. On the contrary, the group A hcAb clones that showed weak inhibition of RBD-ACE2 interaction (1.16 and 2.11) had a deficient virus neutralization (**Figure 3C**). Lastly, the group B 1.29 and 2.20 hcAbs inhibited >90% SARS-CoV-2 infection only at 50 nM (**Figure 3C**), as seen with the Spp pseudovirus. The hcAb 1.29 showed slightly better neutralization of SARS-CoV-2 than 2.20, which was consistent with its greater inhibition of RBD-hACE2 interaction (**Figure 3A**). As for the Spp assay, no inhibition of SARS-CoV-2 infection was observed with a control (C) hcAb (**Figure 3C**).

### hcAb Protection of hACE2-Transgenic Mice Infected With a Lethal Dose of SARS-CoV-2

**Figure 4:**
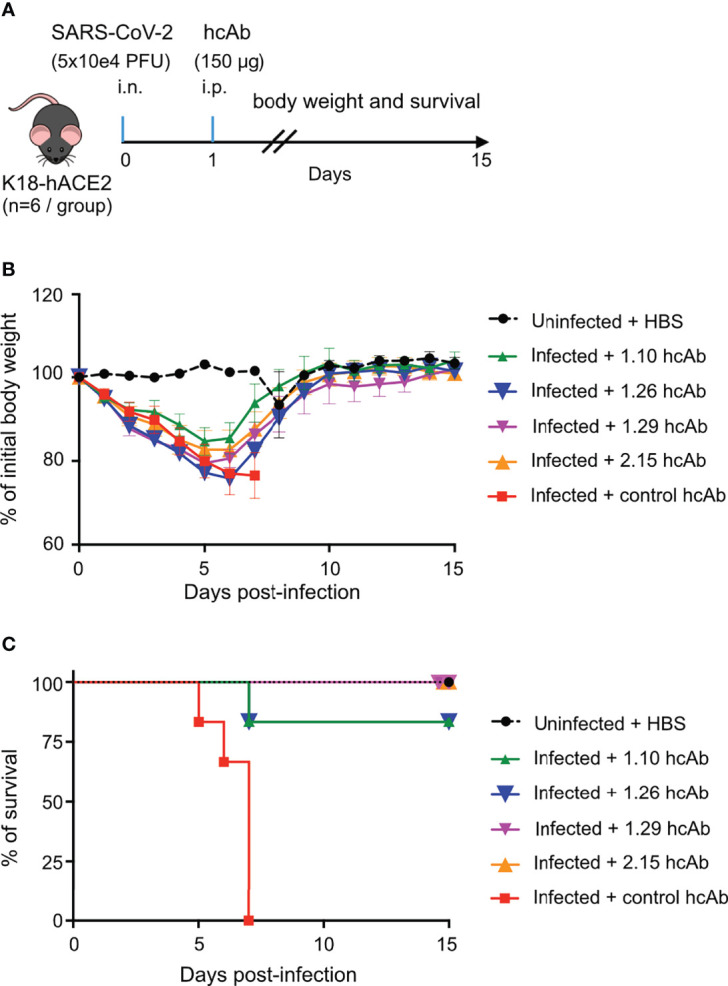
hcAbs protection of hACE2-trangenic mice after a lethal SARS-CoV-2 infection. **(A)** *In vivo* experiment design. On day 0, groups of K18-hACE2 mice (n=6/group) were either infected intranasally (i.n) with a lethal dose of 5x10^4^ plaque forming units (PFU) of SARS-CoV-2 (infected groups) or mock infected with PBS (uninfected group). On day 1 postinfection, 150 μg of 1.10, 1.26, 1.29, 2.15 or control hcAbs, were administered intraperitoneally (i.p.) to animals in the infected groups. The uninfected group was treated i.p. with HBS. **(B)** Percentage of daily body weight of animals in each experimental group (as indicated) relative to their body weight on day 0, before infection. Mean and SD of 6 animals. **(C)** Percentage of daily mice survival in each experimental group up to 15 d.p.i.

Based on hcAb inhibition of RBD-hACE2 interaction and neutralization of SARS-CoV-2 *in vitro* (**Figure 3**), we selected three group A hcAbs (1.10, 1.26 and 2.15) and the hcAb 1.29 from group B to determine their therapeutic efficacy in an animal model of SARS-CoV-2 infection. We performed a post-exposure protection assay in K18-hACE2 transgenic mice that reproduce major clinical symptoms of COVID-19 in humans (56). Groups of K18-hACE2 mice (n=6) were infected intranasally (i.n.) with ~5×10^4^ PFU of B.1.610 lineage SARS-CoV-2 (Materials and methods), or mock infected with sterile PBS. One day post-infection (d.p.i.) each experimental group of mice received the 1.10, 1.26, 2.15, 1.29, or a control hcAb, intraperitoneally (i.p.) in a single dose of 150 μg/mouse (**Figure 4A**). This dose is equivalent to ~8 mg/kg (for a mouse of 18 g), which is in the dose range of most therapeutic Abs administered systemically in humans (from 3 to 20 mg/kg) (57). As control, the uninfected group was also mock-treated i.p. with sterile HBS.

The hcAb did not prevent weight loss of the infected animals in this stringent challenge model, when compared to uninfected controls (**Figure 4B**). All infected groups showed a clear decline in body weight until 5 or 6 d.p.i., with 1.10-treated mice having the less severe reduction in weight compared with uninfected controls. Nonetheless, the four anti-RBD hcAbs significantly reduced lethality (**Figure 4C**), in contrast to control hcAb-treated animals (Mantel-Cox log-rank test for survival *P*<0.0001 between control and 1.10, 1.26, 1.29 and 2.15 hcAbs). All infected mice treated with the control hcAb died between 5 and 7 d.p.i., whereas none died in the groups treated with 1.29 and 2.15, and only one animal out of 6 deceased (~16.6%) in the groups treated with 1.10 or 1.26 at 7 d.p.i. (**Figure 4C**). Notably, all the mice that survived the infection reached the body weight of the uninfected controls 15 d.p.i. (**Figure 4B**), so that they recovered from the disease. Thus, the four RBD-binding hcAbs prevented the lethality of a SARS-CoV-2 infection in K18-hACE2 mice, suggesting their therapeutic potential to reduce the risk of death by COVID-19.

### Structure of the Trimeric S Protein in Complex With RBD-Specific Nbs

**Figure 5:**
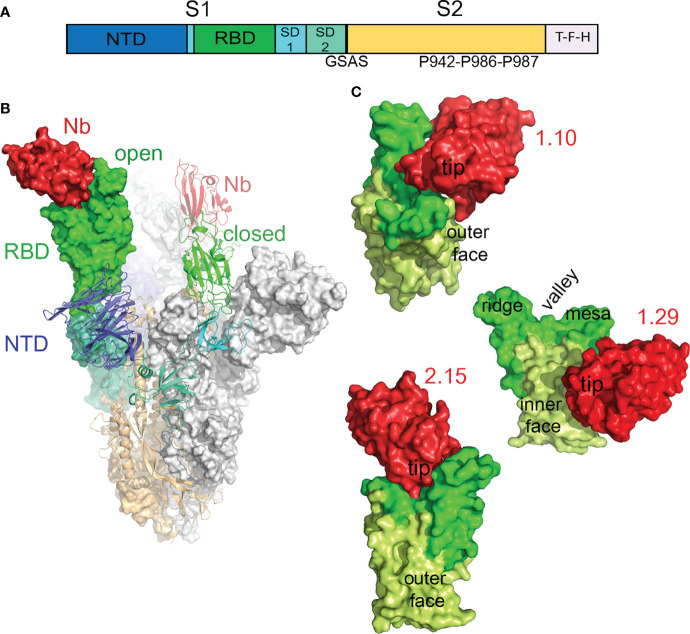
Cryo-EM structure of the SARS-CoV-2 S in complex with RBD-specific Nbs. **(A)** Scheme of the S construct used to generate the S-Nb complexes. The extracelular S1 with the N-terminal domain (NTD), receptor-binding domain (RBD), subdomains 1 and 2, and the S2 region are shown colored. The soluble S contained a T4 trimerization domain (T), a Flag peptide (F), and a 6xHis-tag (H) at its C-termininal end. The furin site was substituted by the GSAS sequence and the indicated three prolines were introduced at the S2 region to enhance protein stability and expression. **(B)** Cryo-EM structure of the trimeric S in the prefusion form with a Nb (2.15) bound to its RBD. Models of the S and the Nb were fitted into the cryo-EM map of the S-2.15 complex (**Figure S6**), as described in Materials and methods. The S monomers with the open RBD are represented as surfaces, either with the domains colored as in A or in grey, whereas the monomer with the closed RBD is shown as ribbon with the domains colored. The two modeled Nbs bound to the RBDs are shown in red. **(C)** Structures of the SARS-CoV-2 neutralizing 1.10, 1.29 and 2.15 Nbs bound to the RBD. Surface representations of RBD-Nb modeled in the cryo-EM maps shown in **Figure S6**; the RBDs are shown with the RBM in green, the RBD core in light-green and the Nbs in red. The RBD surfaces facing toward (inner) or opposity (outer) to the S trimer center, and the RBM regions are indicated.

Next, we used cryo-electron microscopy (cryo-EM) to determine the Nb binding mode to the RBD in a trimeric soluble S protein without the furin cleavage site and stabilized with a T4 phage trimerization domain, as well as with three proline residues in the S2 portion that enhanced protein expression and maintained its prefusion conformation (51) (**Figure 5A**). We prepared S-Nb complexes with the 1.10, 1.26, 1.29 or 2.15 Nbs that showed *in vivo* efficacy against SARS-CoV-2, which were purified for EM data collection (Materials and methods). Subsequently, we carried out particle classification and S-Nb complex reconstruction (**Figures S6A, B**), which gave maps at resolutions ranging from 3.2 to 3.9 Å (**Figure S6C**). Nonetheless, there were marked differences on the map local resolutions between the S1 and S2 regions (**Figure S6B**). The open RBDs exhibited high flexibility and certain loops of the NTD and RBD were not properly defined in the EM maps.

In the timeric complex structures, the S in the prefusion state had two monomers with the RBD in the open conformation and the third monomer with the RBD closed (**Figure 5B**), a form that cannot engage the hACE2 receptor. S-Nb structures showed density corresponding to Nbs attached to the RBDs in the two conformations (red in **Figure S7**). The S-1.29 and S-2.15 reconstructions showed good densities for Nbs bound to the closed RBD and to one open RBD, but poor density to the second open RBD (**Figure S7**), so that, we only modeled two 1.29 and 2.15 Nbs bound to the S (**Figure 5B**). In the S-1.10 complex structure, the Nbs were poorly defined in the map because of the relatively low number of particles used in the reconstruction, likely due to S disruption upon Nb binding; nonetheless, we modeled and refined the Nb bound to one open RBD. The S-1.26 complex reconstruction also showed weak density corresponding to the bound Nb at the top of the RBM (**Figure S7B**); thus, we avoided 1.26 Nb fitting because of its binding mode looked like similar to 2.15.

### Modes of Nb Binding to the SARS-CoV-2 RBD That Mediated Virus Neutralization

Modeling of the 1.10, 1.29 and 2.15 Nbs bound to the SARS-CoV-2 S showed three distinct ways of targeting the RBD that prevented virus infection (**Figure 5C**). Neutralizing 1.10 and 2.15 Nbs from group A recognized the RBM, similar to class 2 human Abs (**Figure 5C**), which bind the RBM in the RBD open and closed conformations (14). Nonetheless, whereas the 2.15 accessed the RBD top, the 1.10 Nb approached the RBM from the RBD outer face (**Figure 5C**), and laid over the NTD. In the 2.15 Nb, the immunoglobulin (Ig) domain tip with the CDRs contacted preferentially the RBM valley and ridge, but the 1.10 bound to the valley and the RBM mesa at the terminal side of the receptor-binding subdomain (**Figure 5C**). The group B 1.29 Nb bound to the edge of the RBD core and to its inner face, in a region that overlaps with that described for class 4 human Abs (**Figure 5C**) (3, 14). Nonetheless, differing from human Abs, the 1.29 Nb interacted through one side of its Ig domain (see below), which run perpendicular to the RBD edge, this binding mode and the small Nb size, allows the single Ig domain to accommodate between closed and open RBDs in the S (**Figure S7C**).

**Figure 6:**
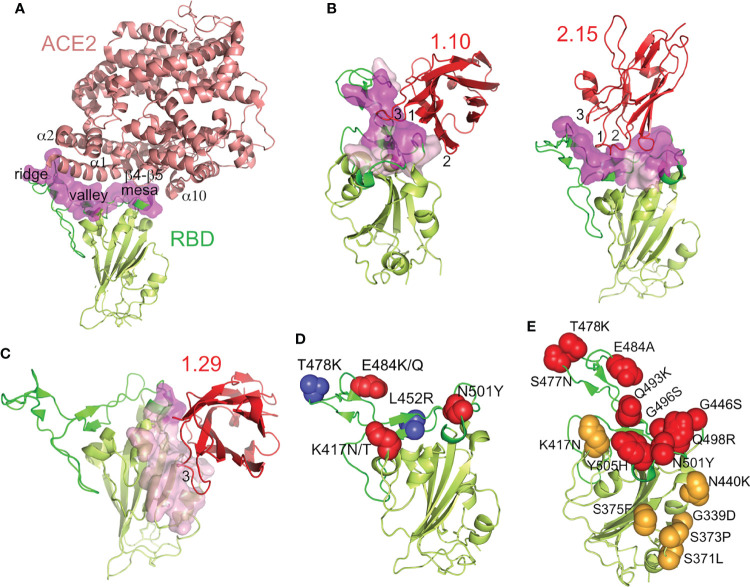
Nb binding epitopes at the SARS-CoV-2 RBD, overlapping with the ACE2 receptor binding region and with mutations in the SARS-CoV-2 RBD variants. **(A)** The SARS-CoV-2 RBD-ACE2 complex structure. Ribbon representation of the RBD-ACE2 crystal structure (PDB id 6LZG), with ACE2 colored in salmon, the RBD with the RBM in green, the core in light-green and with its ACE2-binding residues as a surface in magenta. The ACE2 motifs that contact the indicated RBM regions labeled. **(B, C)** Structures of the SARS-CoV-2 neutralizing Nbs bound to the RBD. Ribbon representations of the RBD and the indicated bound Nb (red) with the CDRs (1, 2 and 3) that contacted the ligand labeled. Surface representations of the RBD residues at the interface with the Nbs are shown pink and with the residues that also engaged ACE2 in magenta, as in panel **(A)**. **(D)** RBD adaptation in SARS-CoV-2 variants of concern, except omicron. RBD residues that changed with respect to WA1 in the alpha (N501Y), beta (K417N, E484K, N501Y), delta (L452R, T478K), gamma (K417T, E484K, N501Y) and kappa (L452R, E484Q) variants are shown as red or blue spheres. **(E)** RBD evolution in the SARS-CoV-2 omicron variant. Side chains of RBD residues altered with respect to WA1 in the RBM or RBD core are shown as red or orange spheres, respectively. Residue substitutions are indicated.

Model fitting into the cryo-EM maps and refinement allowed determination of the Nb binding epitopes in the RBD by analysis of their binding interfaces, as well as its correlation with the hACE2 receptor binding surface in the SARS-CoV-2 RBD (**Figure 6**). The RBD engages hACE2 with the RBM top (**Figure 6A**); the valley cradles the hACE2 α1 helix, its N-terminus and α2 contact the RBM ridge, whereas the RBM mesa region interacts with other hACE2 regions (β4–β5 turn and α10) (11). The RBD region that connects with its receptor largely overlapped the group A 1.10 and 2.15 Nb binding epitopes (magenta in **Figure 6B**). As hACE2, the 2.15 Nb Ig domain held onto the RBM top, and its CDRs made an extensive interaction with the RBM (**Figure 6B**). In the refined Nb-RBD complexes, the Nb CDR1 and CDR2 penetrated into the RBM valley, and the Nb Arg32 and Lys33 residues were closed (2-5 Å) to Glu484 in the RBM ridge (**Figure S8**), whereas the CDR3 bound to the ridge and other Ig domain loops accessed the RBM mesa. Conversely, the 1.10 Nb approached the RBD from its outer face, and its CDR3 laid onto the RBM valley, whereas the other CDRs connected to the mesa (**Figure 6B**).

According to cross-competition experiments (**Figure 1C**), the group B 1.29 Nb had a non-overlapping epitope with the group A Nbs, which was confirmed by the cryo-EM structures (**Figures 6B, C**). This Nb engaged the RBD core, and its epitope was mostly outside the hACE2 biding region. Nonetheless, the bound Nb contacted a few residues at the terminal RBM region (magenta in **Figure 6C**), and its binding configuration likely interfered with RBD docking into hACE2. In contrast to the other Nbs, the 1.29 CDR3 (FG-loop) and the GFC β-sheet of its Ig domain made an extended interaction with the edge of the RBD core and its inner face (**Figure 6C**). The CDR1 and CDR2 of 1.29 were not at the RBD-Nb binding interface.

### hcAb Binding to Prevailing RBD Variants

Circulating SARS-CoV-2 variants of concern contain RBD residue substitutions that increase its hACE2 receptor-binding affinity and enhance virus transmission (22). These substitutions are exclusively at the RBM for the alpha, zeta, kappa and delta variants (e.g., N501Y, E484K/Q, T478K, L452R), whereas beta and gamma contains an additional K417N/T mutation in the RBD core that is close to the RBM (**Figure 6D**). In contrast, the omicron variant contains multiple mutations located both at the RBM as well as the RBD core (**Figure 6E**), which overlapped with the epitopes determined here for the neutralizing Nbs (**Figures 6B, C**).

**Figure 7:**
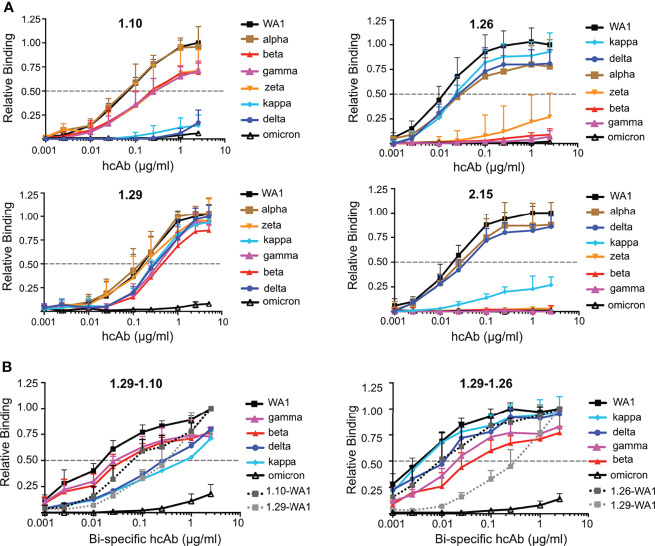
Monospecific and bispecific hcAb recognition of RBD variants. **(A)** Binding of hcAbs 1.10, 1.26, 1.29 and 2.15 (indicated on top of each graph) to RBD-Fc protein variants, which are shown and color coded on the right of each graph; listed from higher (top) to lower (bottom) hcAb binding activity. The OD_490_ determined with serial hcAb dilutions as in **Figure 1** was normalized to the maximum binding signal with the WA1 protein. The dotted line represents half of the maximum hcAb binding to WA1 RBD-Fc, used to determined EC_50_ values. Mean and standard deviation (SD) of at least three independent assays (n ≥ 3). **(B)** Binding of bispecific hcAbs 1.29-1.10 and 1.29-1.26 carried out as in **(A)** Binding of the monospecific hcAbs 1.29, 1.10 or 1.26 to the WA1 RBD are also shown for direct comparison with the bispecific hcAbs. Data are mean and standard deviation (SD) of three independent assays (n = 3).

We evaluated using ELISA group A (1.10, 1.26, 2.15) and group B (1.29) Nbs for binding to the RBDs from variants of concern (alpha, beta, gamma, delta and omicron), as well as the related kappa and zeta variants. To facilitate protein preparation and hcAb binding to plastic-immobilized ligand we used RBD-Fc fusion proteins rather than monomeric RBDs. The RBD-Fc proteins from WA1 strain and from the different virus variants were used to determine binding activity of biotin-labeled hcAbs (**Figure 7A**). The four hcAbs bound similarly to the alpha and WA1 RBDs, indicating that the RBD N501Y substitution did not affect binding of the hcAbs tested. In contrast, the single RBD E484K substitution in the zeta variant severely impaired 1.26 binding and completely abolished 2.15 recognition (**Figure 7A**); this residue is within the epitopes recognized by these Nbs (**Figure 6**), and residue E484 interacted with the basic 2.15 CDR1 in our modeled interaction (**Figure S8**). Likely, this is a key contact for the 2.15 Nb recognition of the RBM, as this Nb did not bind to RBD variants with the E484K (beta, gamma or zeta) or poorly to kappa with E484Q (**Figure 7A**). The 1.26 Nb recognized weakly the RBDs with K484, but it bound the kappa variant that bears Q484 (**Figure 7A**), which indicated that the RBD E484 is not essential for 1.26 binding. In contrast to the 1.26 and 2.15 hcAbs, 1.10 bound to the variants with the E484K substitution (beta, gamma or zeta) (**Figure 7A**), but failed to bind to delta or kappa variants that shared the L452R mutation at the Nb epitope according to structural data (**Figures 6B, D**). Importantly, the RBD mutations L452R and T478K found in the prevalent delta variant did not affect 1.26 or 2.15 hcAb binding to the RBD (**Figure 7A**). However, none of the group A hcAbs bound to omicron RBD (**Figure 7A**). The group B hcAb 1.29, which bound outside the RBM (**Figure 6C**), was able to recognize all RBD variants except omicron (**Figure 7A**). Notably, the binding activity of 1.29 to alpha, zeta, beta, gamma, delta and kappa variants was similar to WA1 RBD or only moderately reduced (ca. 2 to 3-fold) according to EC_50_ values (**Figure 7A**). In contrast, the 1.29 hcAb binding activity to omicron was weak, decreasing at least 100-fold with respect to the WA1 RBD (**Figure 7A**). Contrary to other variants, omicron RBD core mutations S371L-S373P-S375F overlapped with the 1.29 epitope (**Figure 6D**). Analysis of omicron recognition by the other hcAbs of our panel (**Figure 1**) showed absence of binding by all group A hcAbs, and a weak RBD recognition by the other group B 2.20 hcAb (**Figure S9**), similar to 1.29 hcAb.

### Bispecific hcAbs With Increased Affinity for RBD Variants

To increase the binding activity of hcAbs to RBD variants, we evaluated bispecific hcAbs combining group B (1.29) and group A (1.10 or 1.26) Nbs in tandem, fused to the Fc region (**Figures S2C, D**). Biotin-labeled bispecific hcAbs 1.29-1.10 and 1.29-1.26 were tested for binding to RBD-Fc molecules of WA1, beta, gamma, delta, kappa and omicron variants (**Figure 7B**). The 1.29-1.10 hcAb showed greater binding to the WA1, beta and gamma RBDs than the monospecific 1.10 or 1.29 hcAbs, and similar EC_50_ to kappa and delta variants as the 1.29 hcAb (**Figure 7B** left panel). The bispecific 1.29-1.10 hcAb binding to omicron RBD was slightly increased, but still showed weak signals at high concentrations (**Figure 7B** left panel). Similarly, the 1.29-1.26 hcAb improved RBD variant recognition with respect to the monospecific hcAbs (**Figure 7B** right panel). Importantly, except for omicron, this bispecific hcAb EC_50_ for the variants were lower than the 1.29 EC_50_ with the WA1 RBD. Since the 1.29 hcAb molecule was sufficient to provide full protection against lethal SARS-CoV-2 WA1 infection *in vivo*, these bispecific hcAbs, and particularly 1.29-1.26, are promising molecules for therapies against all current SARS-CoV-2 variants except omicron, which escapes from this panel of neutralizing hcAbs.

## Discussion

In an effort to develop therapeutic tools to prevent the COVID caused by SARS-CoV-2 infection, we generated RBD-specific Nbs following camel immunization, and engineered hcAbs molecules that prevented SARS-CoV-2-associated lethality in animal models. The nine Nbs selected and characterized in this study have unique sequences and CDRs (**Figure 1A**). As expected by their distinct immunization origin, none of these CDRs sequences are found in other Nbs reported against the SARS-CoV-2 RBD, despite similarities in their binding modes (see below). Among the four potent neutralizing Nbs tested *in vivo* as hcAbs, we identified SARS-CoV-2 variant-specific molecules, as well as a Nb (1.29) that recognized a conserved RBD site in all variants of concern but in omicron. Combination of Nbs in bispecific hcAbs enhanced variant recognition and resulted in molecules that can bind very efficiently to most RBD variants (EC_50_ ~200 pM), with potential to neutralize diverse SARS-CoV-2 infections.

The RBD is a key domain for CoV cell entry and the main target of CoV-neutralizing Abs (4, 12, 13). RBD immunization facilitates the generation of Ig molecules that neutralized the SARS-CoV-2, such as shown here by the development of camel-derived Nbs. In addition, the use of monomeric RBD facilitated the selection of bacterial-displayed molecules with S or RBD-binding affinities in the low nM or subnanomolar range, comparable to those reported for human Abs (2, 3, 14) and other SARS-CoV-2 RBD-specific Nbs selected from different libraries (42–44). In addition, single domain VHH can be easily linked in tandem to generate multivalent molecules with a single or varied specificities, and with increased antigen-binding avidity (29). This Nb functionality was exploited here to generate bispecific molecules that recognized the RBD of multiple SARS-CoV-2 variants. Multivalent or multispecific Nb/hcAb molecules improving binding to SARS-CoV-2 variants have also been reported recently (35, 41, 42).

Based on S binding cross-competition, we clustered the RBD-specific Nbs selected here from two immunized camels in two different groups. The group B 1.29 and 2.20 Nbs, each coming from a different camel, bound to overlapping RBD epitopes outside of the RBM. Nonetheless, the structures of group A Nbs (1.10, 1.26 and 2.15) showed that they bound to the RBM, and to residues engaged in hACE2 recognition (**Figure 6**). The 1.26 and the 2.15 Nbs approached to the RBM top, and their footprints largely overlapped with the receptor binding region, as described for class 2 human Abs or Nbs such as Ty1 (14, 38) (**Figure S10**). Differences among the binding interactions of these Nbs are likely due to their distinct CDRs. The 2.15 Nb Ig tip and its CDRs contacted the RBM valley and ridge, and the basic 2.15 CDR1 interacted with E484 at the ridge; consistently, the E484 substitutions to Lys or Gln prevented Nb binding. The 1.10 Nb is distinct from 2.15 or Ty1, as it did not access the RBD from the top, but on its outer face, with the Nb tip connected with the RBM valley and the mesa rather than the ridge. This agree with 1.10 Nb binding to RBD variants bearing substitutions at E484 (**Figure 7**), and the lack of binding to RBDs that contained the L452R mutation at the RBM valley (**Figures 6**, **7**). Cryo-EM samples prepared with the S-1.10 complex resulted in low number of S trimers and the formation of S protein aggregates, perhaps due to Nb-mediated S protein disassemble. Nb binding through the RBD outer face and close to the NTD of a different monomer could weaken intermonomer interactions and facilitate S1 dissociation from S2, such as described by multivalent Nb binding to the S (41).

In contrast to the other Nbs analyzed here by cryo-EM, the 1.29 Nb bound outside the RBM and it was not affected by most substitutions in SARS-CoV-2 variants, except omicron, which accumulates multiple substitions in the RBD core (**Figure 6D**). The Nb Ig of 1.29 laid perpendicular to the RBD core β-sheet and its CDR3 interacted with the inner face, on a conserved region in sarbecoviruses that is also recognized by class 4 human Abs (42). Nonetheless, this RBD recognition mode, which engaged the Nb Ig domain GFC β-sheet raher than the Ig domain tip, is distinct from SARS-CoV-2 human Abs, but very similar to other reported Nbs (Nb30 or VHH V) (**Figure S10**) (41, 42) that we clustered here as group B Nbs. They are a valuable class of molecules to mediate efficient SARS-CoV-2 neutralization, either alone or in combination with RBM-binding Nbs (**Figure 7**), which generated bispecific molecules with enhanced virus neutralization and reduced mutational escape (41). The 1.29 Nb epitope appears an important Nb-specific antigenic subsite at the SARS-CoV-2 RBD, outside the receptor-binding region. Nonetheless, the emergence of the omicron variant with multiple substitions around this Nb-recognition site is currently challenging the efficacy of group B neutralizing Nbs or class 3/4 Abs that bind outside the RBM.

Most of the RBD-specific hcAbs analyzed here blocked RBD binding to hACE2 and neutralized SARS-CoV-2 in cell cultures (**Figure 3**), indicating that they prevented virus cell entry. Among the SARS-CoV-2 neutralizing hcAbs *in vitro*, we analyzed the therapeutic potential of four selected molecules that targeted different RBD sites in hACE2 transgenic mice. All the animals infected with a lethal dose of the SARS-CoV-2 and treated with the 1.29 or 2.15 hcAbs by 24 h.p.i. recovered from the infection, whereas the administration of the 1.10 or 1.26 hcAbs rescued ~84% of the animals from COVID death, which reached a body weight like uninfected control animals at the end of the experiment. Depite recovery, we observed important weight loss in all infected mice with the high viral titer administered in the *in vivo* assay (5x10^4^ PFU). This weight loss could perhaps be reduced by the earlier administration of the hcAbs. Even though, the four hcAbs tested showed a good efficacy to prevent animal death by SARS-CoV-2 infection, the group B 1.29 hcAb that bound outside the RBM was as efficient as the group A Nbs with higher S binding affinity and better SARS-CoV-2 neutralization in cell cultures, which indicated the importance of *in vivo* studies to determine the best candidates for SARS-CoV-2 therapy. In this regard, fusion to the Fc region in hcAbs is a key factor for their therapeutic efficacy *in vivo* against SARS-CoV-2 infections due to their recognition by Fcγ receptors, as it has been demonstrated with human therapeutic Abs (58). In addition, our results suggested that bispecific (or multispecific) hcAb molecules combining Nbs against non-overlapping RBD epitopes can be excellent therapeutic candidates for the treatment of the COVID-19 caused by SARS-CoV-2 variants. Nonetheless, the new omicron variant escaped from the recognition of multiple hcAb molecules binding diverse RBD epitopes that we isolated from animals immunized with the WA1 strain included in most vaccines. These data presumed induction of reduced omicron-neutralizing humoral immune responses by the current vaccines, as well as the failure of approved SARS-CoV-2 therapeutic Abs that target the RBD. Likely, only omicron-specific Abs and vaccines will have the desirable therapeutic or profilactic capabilities to prevent this variant infection, which urges the update of current therapies to overcome the COVID-19 pandemic.

## Data Availability Statement

The original contributions presented in the study are included in the article/**Supplementary Material**. Further inquiries can be directed to the corresponding authors.

## Ethics Statement

The dromedary camel immunization protocol followed European Union and Spanish guidelines of animal experimentation and was approved by the Ethics Committee for Animal Experimentation from University of Las Palmas de Gran Canaria and the Consejería de Agricultura, Pesca y Aguas of the Canary Islands Goverment (reference OEBA-ULPGC 01-2020 R2). Experimentation with mice was conducted in the biosafety level 3 facilities at Centro de Investigación en Sanidad Animal at Instituto Nacional de Investigación y Tecnología Agraria y Alimentaria (CISA-INIA, CSIC). Experiments were approved by the Ethical Committee of Animal Experimentation of INIA and by the Division of Animal Protection of the Comunidad de Madrid (PROEX 115.5-21). Animals were handled in strict accordance with the guidelines of the European Community 86/609/CEE.

## Author Contributions

LF and JMC conceived and supervised this study. JAC performed camel immunization and blood sample collection. YM constructed and screened the Nb libraries. YM, MN, MG, and JMC produced proteins and carried out binding assays and determination of affinities. YM and MC performed Ab cross-competition and RBD-hACE2 competition experiments. PG and UG performed *in vitro* virus neutralization assays. NJO, JS, and MM-A performed the *in vivo* protection assays. MN and JMC prepared samples for cryo-EM. RA collected data. RM carried out particle reconstruction and JMC achieved model building and refinement. LF and JMC analyzed all data, wrote the manuscript and prepared the final Figures. All authors contributed to the article and approved the submitted version.

## Funding

This work was partially funded by *Ministerio de Ciencia e Innovación* (MICIN; https://www.ciencia.gob.es/) and the Spanish Research Council (CSIC; https://www.csic.es/) under grants PIE-RD-COVID 19 (No 202020E079) and PTI+ Salud Global REC_EU (No SGL 2103051, NextGenerationEU) to LF, JMC, PG, and UG, and (No SGL 2103053, NextGenerationEU) to MM-A. This study was partially conducted within the CSIC Antiviral Screening Network, an infrastructure supported by NextGeneration EU funds (https://ec.europa.eu/info/strategy/recovery-plan-europe_es) from the European Union and the European Virus Archive Global (EVag) of the European Union’s Horizon 2020 (https://ec.europa.eu/programmes/horizon2020/en/home) research and innovation programme (No 871029) to PG and UG. EM facilities of CNB-CSIC were supported by *Ministerio de Ciencia e Innovación* (MICIN; https://www.ciencia.gob.es/), EU-FEDER (https://ec.europa.eu/regional_policy/es/funding/erdf/) CRIOMECORR project (ESFRI-2019-01-CSIC-16). JMC access to the European Synchrotron Radiation Facility (ESRF) CM01 line through the Iberian-BAG, and to the Instruct Image Processing Center (I2PC, http://i2pc.es/) by projects PID16168 and PID14989. The funders had no role in study design, data collection and analysis, decision to publish, or preparation of the manuscript.

## Conflict of Interest

JMC, YM, MN, PG, UG, JS, MM-A, JAC, and LF are co-inventors on patent applications covering the Nb and hcAb molecules described in this manuscript.

The remaining authors declare that the research was conducted in the absence of any commercial or financial relationships that could be construed as a potential conflict of interest.

## Publisher’s Note

All claims expressed in this article are solely those of the authors and do not necessarily represent those of their affiliated organizations, or those of the publisher, the editors and the reviewers. Any product that may be evaluated in this article, or claim that may be made by its manufacturer, is not guaranteed or endorsed by the publisher.
