# Supplementary material for: Nanobodies Protecting From Lethal SARS-CoV-2 Infection Target Receptor Binding Epitopes Preserved in Virus Variants Other Than Omicron
Source: Front Immunol. 2022 Apr 25;13:863831. doi: 10.3389/fimmu.2022.863831 (PMC9082315; doi:10.3389/fimmu.2022.863831)
Supplement: Supplementary file 1 [file DataSheet_1.pdf]

Supplementary Material

Nanobodies protecting from lethal SARS-CoV-2 infection target receptor binding epitopes preserved in virus variants other than omicron

José M. Casasnovas[1]*, Yago Margolles[1], María A. Noriega[1], María Guzmán[1], Rocío Arranz[1], Roberto Melero[1], Mercedes Casanova[1], Juan Alberto Corbera[2], Nereida Jiménez-de-Oya[3], Pablo Gastaminza[1], Urtzi Garaigorta[1], Juan Carlos Saiz[3], Miguel Ángel Martín-Acebes[3], and Luis Ángel Fernández[1]*

[1]Departments of Macromolecule Structure, Microbial Biotechnology, and Cellular and Molecular Biology, Centro Nacional de Biotecnología, Consejo Superior de Investigaciones Científicas (CNB-CSIC), Darwin 3, Campus Cantoblanco, 28049 Madrid, Spain.

[2]Instituto Universitario de Investigaciones Biomédicas y Sanitarias (IUIBS). Facultad de Veterinaria. Universidad de Las Palmas de Gran Canaria (ULPGC). Campus Universitario de Arucas, s/n. 35413 Arucas, Gran Canaria, Spain.

[3]Department of Biotechnology, Instituto Nacional de Investigación y Tecnología Agraria y Alimentaria, Consejo Superior de Investigaciones Científicas (INIA, CSIC), Carretera de A Coruña km 7.5, 28040 Madrid, Spain

* For correspondence: jcasasnovas@cnb.csic.es, lafdez@cnb.csic.es

Contents:

- Supplementary Materials and methods
- Supplementary References
- Table S1
- Figures S1 to S10.

Supplementary Materials and Methods

DNA manipulation and cloning procedures.

Plasmid constructions and cloning procedures were performed following standard protocols of DNA manipulation, restriction and ligation. The genes coding for the S gene and RBD variants of SARS-CoV-2 were chemically synthesized with codon optimization for expression in mammalian cells (GeneART, ThermoFisher Scientific). Plasmid constructs for protein expression and VHH cloning are described in the corresponding sections. Oligonucleotide primers were ordered from Merck-Sigma. Plasmid constructs were sequenced by the chain-termination Sanger method (Macrogen Inc.).

Growth and induction of bacteria for nanobody surface display.

In the case of single clones, individual colonies grown on LB-Cm-Glu plates were used to start preinocula cultures (10 ml) grown overnight (o/n) under static conditions in liquid LB-Cm-Glu. In the case of immune libraries, or RBD-selected sublibraries, sufficient clones to cover at least 10 times their clonal diversity were harvested from bacterial lawns grown on LB-Cm-Glu plates. Initial colony forming units (CFU) plated on bacterial laws were determined by parallel plating of serial dilutions on LB-Cm-Glu plates. Bacteria grown as lawns on plates were harvested in LB-Cm-Glu and used to start preinocula cultures (50 ml) of libraries, by dilution to an OD_{600} of 0.5 and grown o/n under static conditions in the same media. For induction, bacteria were harvested from the o/n static cultures by centrifugation (3000 xg, 5 min), inoculated to an initial OD_{600} of 0.5 in LB-Cm with 0.05 mM IPTG (10 ml for individual clones and 50 ml for immune libraries), and incubated for 3 h with agitation (170 rpm) at 30 °C.

Selection of RBD-binding Nbs from immune libraries displayed on *E. coli*.

About $5x10^9$ CFU of bacteria (5 OD_{600} unit) were harvested by centrifugation (3000 xg, 5 min) from induced cultures representing each library (D1 and D2, as described above). Harvested bacteria were washed 2 times with 5 ml of phosphate buffered saline (PBS; 8 mM Na_2HPO_4, 1.5 mM KH_2PO_4, 3 mM KCl, 137 mM NaCl, pH 7.0) at room temperature

(RT) and resuspended in 1 ml of PBS supplemented with 0.5% (w/v) of bovine serum albumin (PBS-BSA). For every library, duplicated 100 µl aliquots of this bacterial sample (~5x10^8 CFU) were mixed in a total volume of 200 µl with biotinylated RBD in the same buffer at various final concentrations depending on the selection step. For the initial magnetic cell sorting (MACS) step, 100 nM biotinylated RBD was used. After 1 h of incubation at RT, bacteria were washed with 2 ml of PBS (2 times) and resuspended in 180 µl of PBS-BSA and 20 µl of anti-biotin paramagnetic beads (Miltenyi Biotec). After 20 min of incubation at 4 °C, bacteria were again washed (2 times in PBS), resuspended in 500 µl of PBS-BSA and loaded onto a MACS MS column (Miltenyi Biotec) that had been previously equilibrated with 500 µl of PBS-BSA and placed on an OctoMACS Separator (Miltenyi Biotec). The columns were washed 3 times with 500 µl of PBS-BSA to remove unbound bacteria. After that, MACS column was removed from the separator and placed onto a collector tube for elution of the column-bound bacteria with 2 ml of LB-Cm-Glu. The eluted fractions were plated as a lawn large p150 LB-Cm-Glu agar plates (0.3 ml/plate) and grown at 30 ºC. In parallel, serial dilutions were plated in p60 LB-Cm-Glu agar plates for CFU counting. For the subsequent selection cycle, bacteria from each library were harvested from lawns, grown and induced independently, and subjected to a second MACS as above but using 50 nM of biotinylated RBD. Bound bacteria from this second MACS cycle were plated, harvested, grown and induced for fluorescence activated cell sorting (FACS).

For FACS, induced bacteria were washed and resuspended in PBS-BSA as above, and 100 µl (~5x10^8 CFU) were mixed in a total volume of 200 µl with biotinylated RBD (50 nM final concentration) and mouse anti-c-myc monoclonal Ab (20 µl of a 1:50 dilution of 9B11 clone, Cell Signaling, Ref: 2276). After 1 h incubation at RT, bacteria were washed 3 times with 1 ml of PBS and resuspended in 200 µl final volumen of PBS-BSA containing appropriated dilutions of secondary fluorophore-labelled reagents: Goat anti-mouse IgG-Alexa Fluor 488 conjugated polyclonal Ab (1:500; ThermoFisher Scientific, Ref: A11029) and Streptavidin-Allophycocyanin (APC) (1:100; SouthernBiotech, Ref: 7105-11). After 1

h incubation at 4 °C (in the dark), bacteria were washed 3 times and resuspended in 0.5 ml of PBS for sorting in a FACS vintage SE cytometer (Becton Dickinson). At least $1x10^6$ bacterial events were processed per sample. Double-stained bacterial population with high fluorescence intensity signals was collected (at least ~$5x10^3$ events) in a sterile tube containing 2 ml of liquid LB-Cm-Glu medium and plated. The bacterial pool recovered from the FACS cycle was plated on LB-Cm-Glu agar and serial dilutions were used to isolate individual colonies for clonal analysis.

Protein labelling.

Purified proteins (i.e., RBD, RBD-Fc, trimeric S, hcAbs, or bispecific hcAbs) and human fibrinogen (Enzyme Research Laboratories) were covalently labelled with biotin using biotinamidohexanoic N-hydroxysuccinimide ester (biotin-NHS, Sigma, Ref: B2643). Biotin-NHS was dissolved in dimethyl sulfoxide (DMSO, Sigma Ref: D2438) at 20 mg/ml and immediately used for protein labelling. Proteins (between 0.25 to 1 mg) were incubated with biotin-NHS at a molar ratio of 1:20 (protein:biotin-NHS) in 1 ml HBS for 2 hr at RT. The reaction was blocked by adding Tris-HCl pH 7.5 to a final concentration of 50 mM and further incubated 1 hr at 4°C. Next, free biotin molecules were removed using a Amicon® Ultra-4 centrifugal filter unit (Millipore) of a convenient size cut-off (3 kDa or 10 kDa) by 3 washes in HBS (ca. 5-10 ml), and labelled proteins were concentrated to ~0.5 ml.

Enzyme-linked immunosorbent assays.

Antigen-coated immunoplaes were washed with PBS and blocked with 200 µl/well of 3% (w/v) skimmed milk in PBS at RT for 2 hr, or at 37 °C for 1 h. The indicated dilutions of camel sera or hcAbs were prepared in 1% (w/v) skimmed milk in PBS and added to the wells at RT for 1 hr, or at 37 °C for 30 min. For hcAb competition experiments, a dilution of the biotin-labelled hcAb at 0.1 µg/ml was prepared in 1% (w/v) skimmed milk-PBS containing 20 µg/ml of the indicated unlabelled hcAb competitor, and the mixture was added to S-coated immunoplates for 1 hr at RT. In all cases, after incubation the wells were washed three times with PBS and appropriated dilutions of secondary reagent

conjugated to horseradish peroxidase (HRP) in 1% (w/v) skimmed milk-PBS were added for detection of bound Abs. After 1 hr at RT, or 30 min at 37 ºC, plates were washed with PBS as above, and developed with H_2O_2 and o-phenylenediamine (OPD; Merck-Sigma) as reported previously (1). The Optical Density (OD) at 490 nm (OD_{490}) of plate wells were determined (iMark, Bio-Rad) and the values corrected with the background OD_{490} of wells without antigen or with BSA.

In ELISAs to monitor hcAb binding to RBD variants, biotin-labeled hcAbs were used, and wells were coated with affinity purified RBD-Fc proteins to facility Ab binding to the RBD bound to plastic. Similar amounts of the RBD variants (~2 µg/ml) were used for coating 96-well plates and the actual quantity of plastic bound RBD-Fc proteins for each variant was determined by taking advantage of the HA tag in the constructs using an anti-HA mAb and secondary goat anti-mouse IgG-HRP (ThermoFisherScientific ref. G-21040). Differences in the HA signals were used to adjust the background-corrected OD_{490} acquired with the variants with respect to the WA1 protein.

Supplementary References

1. V. Salema, A. López-Guajardo, C. Gutiérrez, M. Mencía and L. Á. Fernández: Characterization of nanobodies binding human fibrinogen selected by E. coli display. *J Biotechnol*, 234, 58-65 (2016) doi:10.1016/j.jbiotec.2016.07.025

Table S1. Kinetic rate constants (*kass*, *kdis*) and equilibrium dissociation (K_D) constants for Nb and hcAb binding to RBD and S proteins (*).

	RBD			S		
Nb	kass x 10^{-5} (M-1s-1)	kdis x 10^{4} (s-1)	K_D (nM)	kass x 10^{-5} (M-1s-1)	kdis x 10^{4} (s-1)	KD (nM)
1.10	3.45 (0.7)	13.66 (0.6)	3.96 (0.7)	2.73 (0.4)	10.60 (0.6)	3.88 (0.8)
1.16	19.65 (5.8)	70.47 (4.5)	3.59 (0.9)	15.41 (0.8)	84.00 (8.1)	5.45(0.8)
1.26	24.45 (0.3)	6.24 (0.7)	0.26 (0.0)	16.28 (1.9)	4.79 (0.5)	0.29 (0.0)
1.28	2.28 (0.3)	9.85 (3.5)	4.32 (1.0)	5.40 (0.4)	9.59 (1.3)	1.78 (0.1)
1.29	4.76 (1.8)	61.36 (2.8)	12.89 (4.5)	9.44 (1.7)	43.20 (5.6)	4.58 (0.2)
2.1	13.39 (1.9)	23.26 (0.0)	1.74 (0.3)	10.93 (0.7)	22.70 (1.2)	2.08 (0.0)
2.11	22.9 (1.6)	49.69 (1.3)	2.17 (0.1)	20.56 (5.0)	54.70 (5.0)	2.66 (0.4)
2.15	20.18 (0.3)	7.78 (0.3)	0.39 (0.0)	21.62 (1.2)	6.71 (0.5)	0.31 (0.0)
2.20	13.03 (0.8)	10.05 (1.3)	0.77 (0.1)	14.0 (4.4)	11.10 (0.8)	0.80 (0.2)
AVE	13.79	28.04	3.34	12.93	27.50	2.43
hcAb	kass x 10^{-5} (M-1s-1)	kdis x 10^{4} (s-1)	K_D (nM)	kass x 10^{-5} (M-1s-1)	kdis x 10^{4} (s-1)	KD (nM)
1.10Fc	3.09 (0.4)	2.37 (0.3)	0.77 (0.0)	1.60 (0.3)	2.70 (0.2)	1.68 (0.4)
1.16Fc	17.21 (4.0)	4.38 (1.6)	0.25 (0.2)	10.80 (1.6)	2.89 (0.1)	0.27 (0.0)
1.26Fc	17.61 (3.7)	1.48 (0.6)	0.08 (0.0)	10.55 (2.1)	0.58 (0.3)	0.06 (0.0)
1.28Fc	2.47 (0.4)	2.36 (0.1)	0.96 (0.1)	2.13 (0.1)	2.59 (0.5)	1.22 (0.2)
1.29Fc	3.53 (0.4)	2.21 (0.3)	0.63 (0.0)	3.38 (0.2)	1.41 (0.6)	0.42 (0.1)
2.1Fc	7.71 (3.1)	2.81 (0.7)	0.36 (0.1)	4.80 (0.7)	2.18 (1.0)	0.45 (0.2)
2.11Fc	18.23 (7.0)	3.00 (0.6)	0.16 (0.0)	9.03 (1.3)	2.57 (0.1)	0.28 (0.1)
2.15Fc	14.25 (0.2)	1.20 (0.4)	0.08 (0.0)	6.35 (0.0)	2.02 (0.7)	0.32 (0.1)
2.20Fc	10.86 (1.3)	2.33 (0.8)	0.21 (0.1)	3.01 (0.4)	5.14 (1.0)	1.71 (0.6)
AVE	10.55	2.46	0.39	5.74	2.46	0.71

(*) Note: Kinetic rate constants (kass or kdis) determined in the BIAcore from sensorgrams shown in Figures 2 and S4, as described in Materials and methods. Equilibrium dissociation constants (K_D) determined from the kdis/kass ratio. Mean and SD (in parenthesis) of the constants determined in two surfaces (RBD) or two experiments (S). Average of the hcAb and Nb binding constants are also included (AVE).

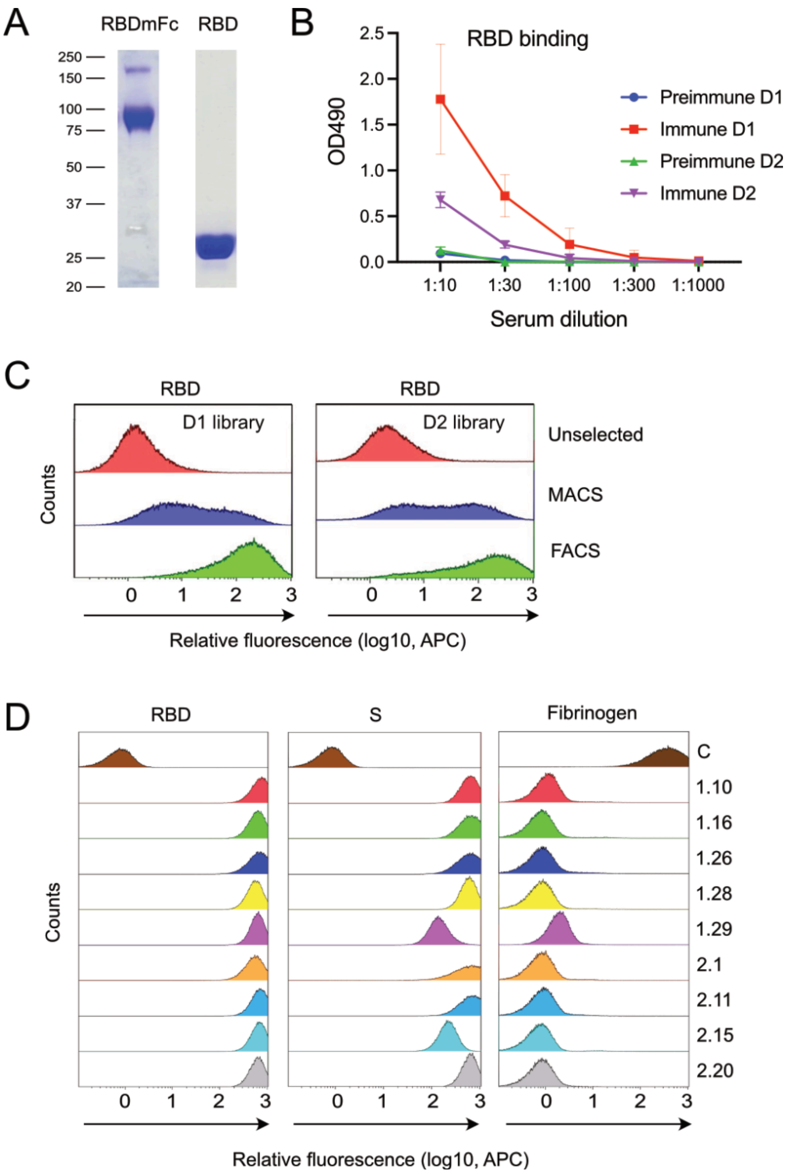

Figure S1. **Selection of nanobodies binding to SARS-CoV-2 RBD by *E. coli* surface display. A.** Proteins used in immunization and nanobody selection. SDS-PAGE (10%) under reducing conditions of purified dimeric RBDmFc and monomeric RBD used for immunization of two dromedaries and for nanobody selection, respectively. Size (kDa) and migration of molecular weight markers are indicated on the left. **B.** Anti-RBD antibody titers in the serum of immunized animals determined by ELISA. Optical density at 490 nm (OD490) determined at different dilution of the sera collected before and 4 days after dromedary D1 and D2 immunization (see Materials and methods). Mean and standard deviation (SD) of three independent assays (n=3). **C.** Enrichment of bacteria displaying Nb libraries generated from dromedaries (D1 and D2) immunized with the SARS-CoV-2 RBD. Flow cytometry of bacterial pools before (unselected) and after selection with monomeric RBD by magnetic cell sorting (MACS) and fluorescence activated cell sorting (FACS). Bacteria were incubated with biotin-labeled RBD (5 nM) and stained with Streptavidin-APC. **D.** Flow cytometry of bacterial clones selected from the D1 (1.10, 1.16, 1.26, 1.28 and 1.29) and the D2 (2.1, 2.11, 2.15 and 2.20) libraries. Bacteria were incubated with the indicated biotin-labeled protein, RBD (5 nM), S (5 nM), or human fibrinogen (100 nM), and stained with Streptavidin-APC. Bacteria displaying a Nb binding to human fibrinogen were used as control (C) (1).

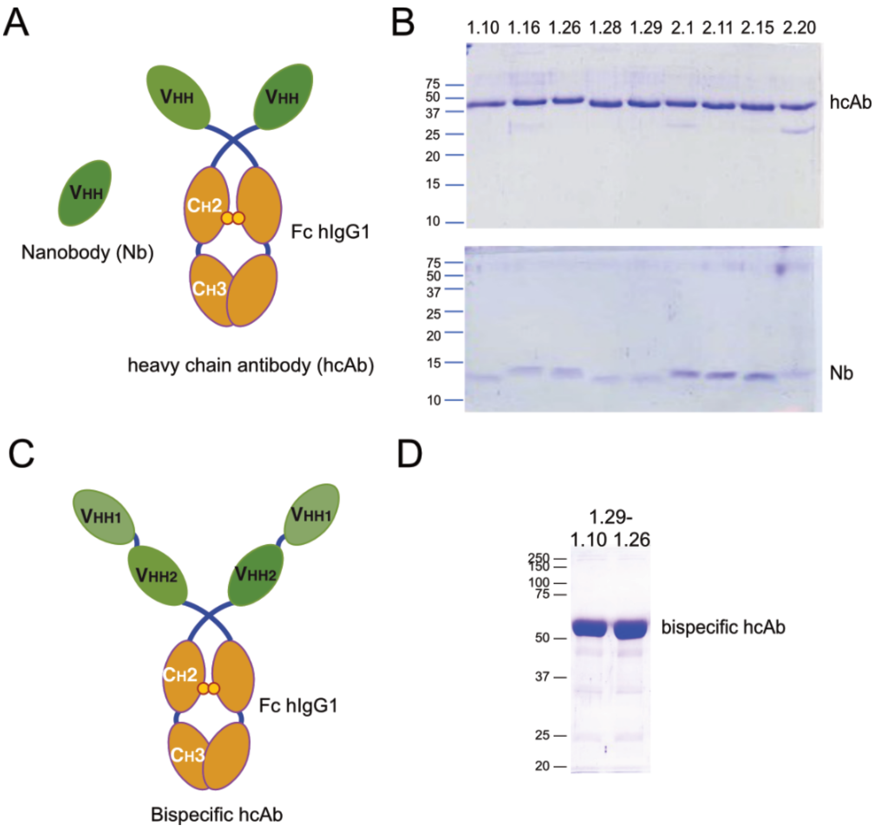

Figure S2. Nb and hcAb proteins prepared in mammalian cells.
A. Graphical representation of single VHH domain or Nanobody (Nb), and of the heavy-chain antibody (hcAb) generated by the fusion of a VHH with the Fc region of human IgG1 (Fc hIgG1). **B.** SDS-PAGE (12%) of purified 1.10, 1.16, 1.26, 1.28, 1.29, 2.1, 2.11, 2.15 and 2.20 hcAbs (top) and the Nbs (bottom) generated from the the hcAbs as described in Materials and methods. **C.** Representation of a bispecific hcAb with two distinct VHH domains in tandem and fused to the Fc hIgG1. **D.** SDS-PAGE (10%) of the affinity purified 1.29-1.10 and 1.29-1.26 bispecific hcAbs from transfected cell culture supernatants. In B and D, size (kDa) and migration of molecular weight markers are indicated on the left.

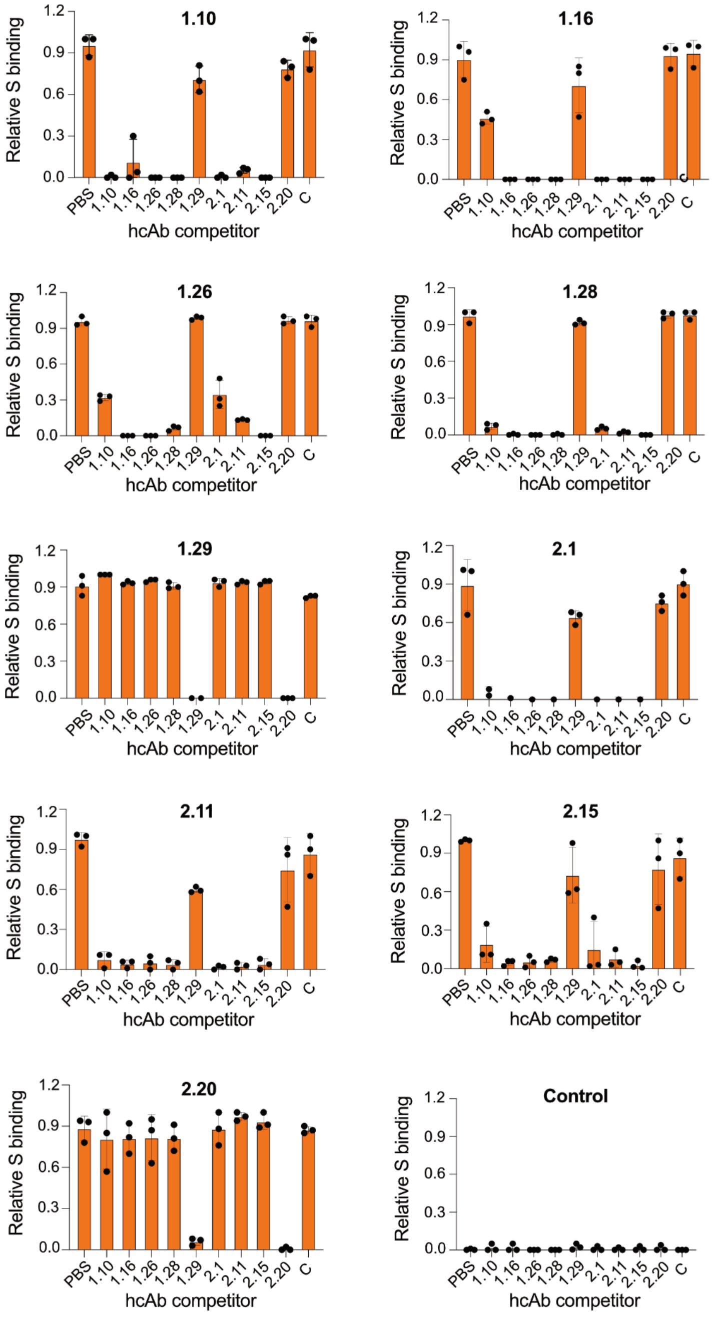

Figure S3. Binding competition between RBD-specific hcAbs. Binding of biotin-labelled hcAb (as indicated on top of each graph) to S, in the presence of 200-fold molar excess of unlabelled hcAb (shown at the x-axis) or without competitor (PBS), determined by ELISA as described in Methods. Binding signals for each hcAb are normalized to the sample without competitor (PBS). Mean and standard deviation (SD) of three independent experiments (n=3).

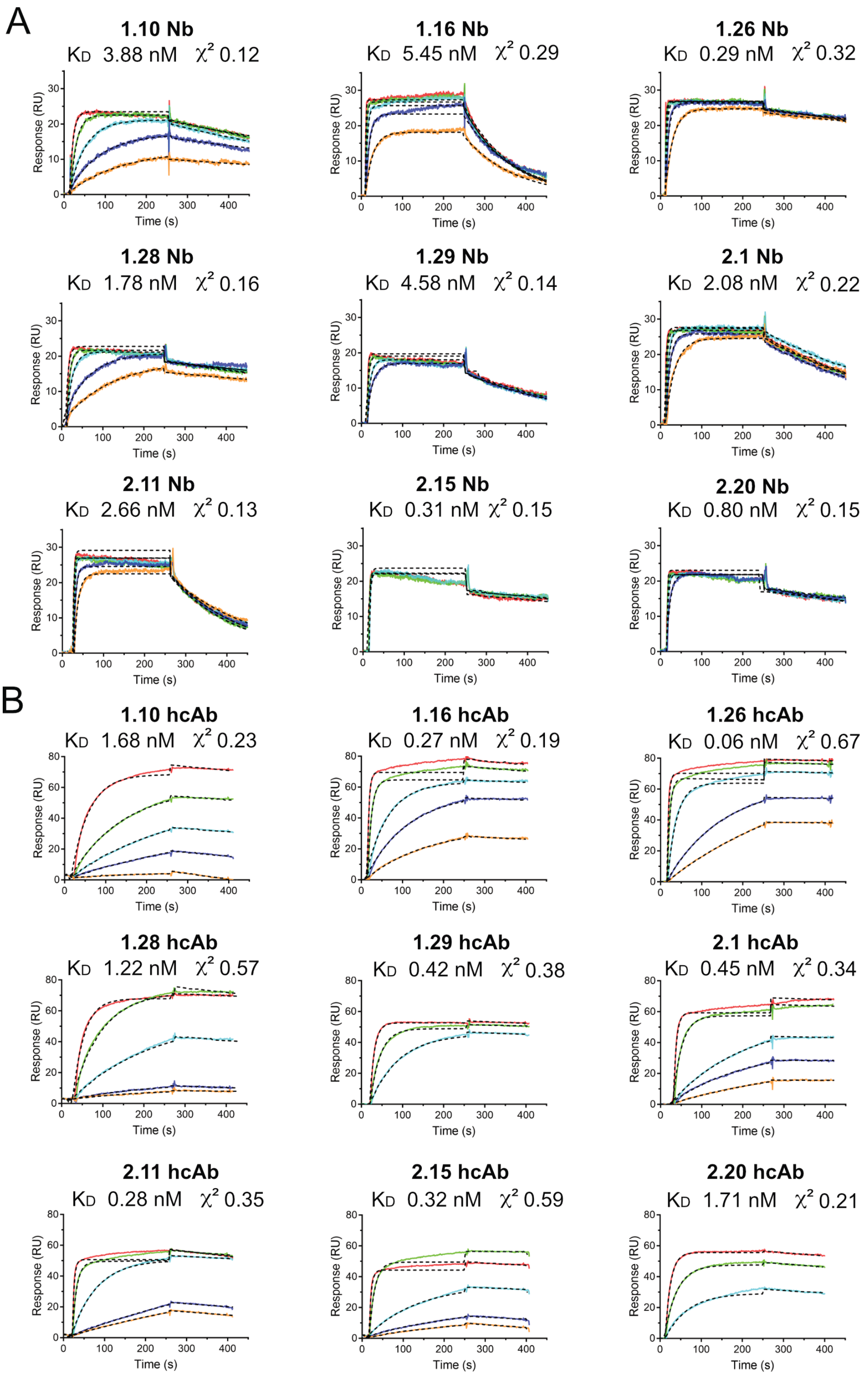

Figure S4. Nb and hcAb binding to the SARS-CoV-2 S ligand in real time. Overlayed sensorgrams recorded during the association and dissociation of the indicated Nbs (**A**) or hcAbs (**B**) through BIAcore sensor chip surfaces with captured S. Nb or hcAb concentrations of 25 to 400 nM or 10 to 200 nM (color coded), respectively, were injected through the surface with S and a control surface. The plots show the specific response (RU) after double referencing (see Materials and methods). The discontinuous dark lines represent the curve fitting to a 1:1 Langmuir model for determination of the kinetic constants, shown in Table S1. The equilibrium dissociation contants (K_D) and the fitting χ^2 are indicated here.

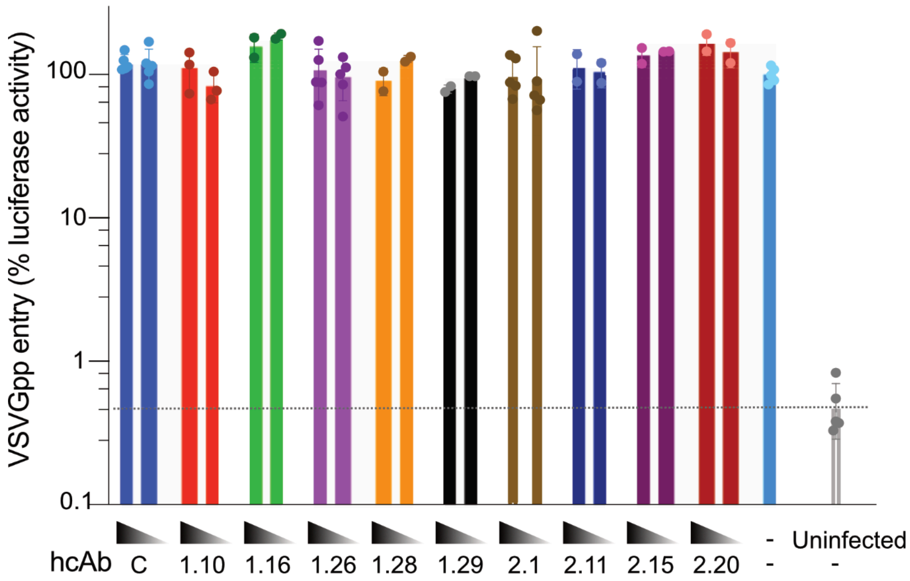

Figure S5. RBD-specific hcAb cell entry inhibition of retrovirus pseudotyped with the Vesicular Stomatitis virus G protein (VSVGpp). Cell entry of luciferase-encoding retroviral particles in Vero-E6 cells was determined from the cell-associated luciferase signal 16 h.p.i. (see Materials and methods). Relative entry of virus particles preincubated with the indicated hcAbs (1.10, 1.16, 1.26, 1.28, 1.29, 2.1, 2.11, 2.15, 2.20) or a control (C) at two protein concentrations (50 and 5 nM), with respect to virus samples without hcAbs (-). Background luciferase activity of uninfected cell cultures is shown with a dashed line. Plotted mean and SD.

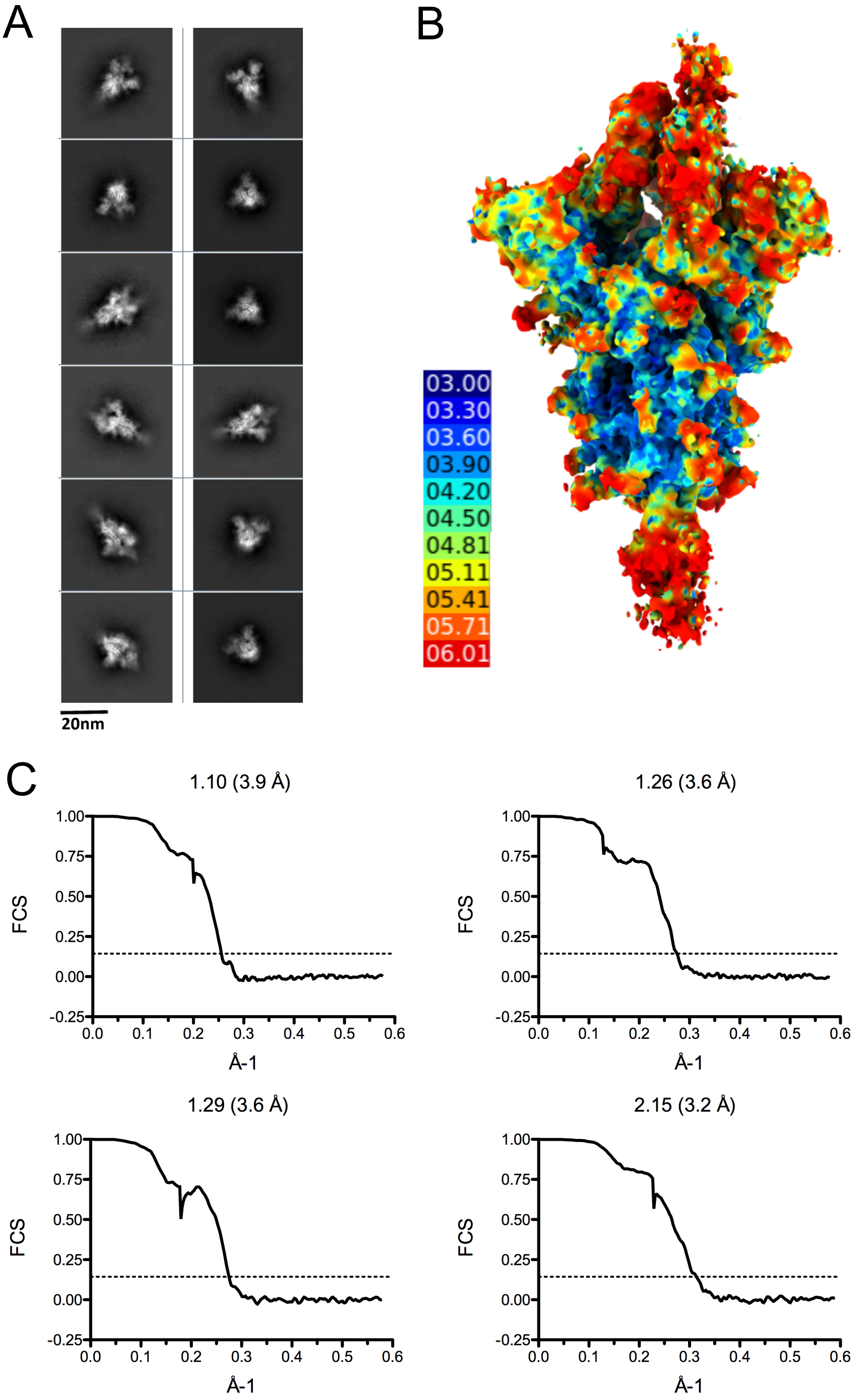

Figure S6. Cryo-EM data processing and S-Nb particle reconstruction. A. Representative 2D average classes. **B**. S-2.15 complex reconstruction and local map resolution. **C.** Fourier Shell correlation (FSC) in the S-Nb particle reconstructions. FSC versus resolution (1/Å), and resolution determination at the 0.143 value for the reconstructions of the S with bound 1.10, 1.26, 1.29 or 2.15 Nbs.

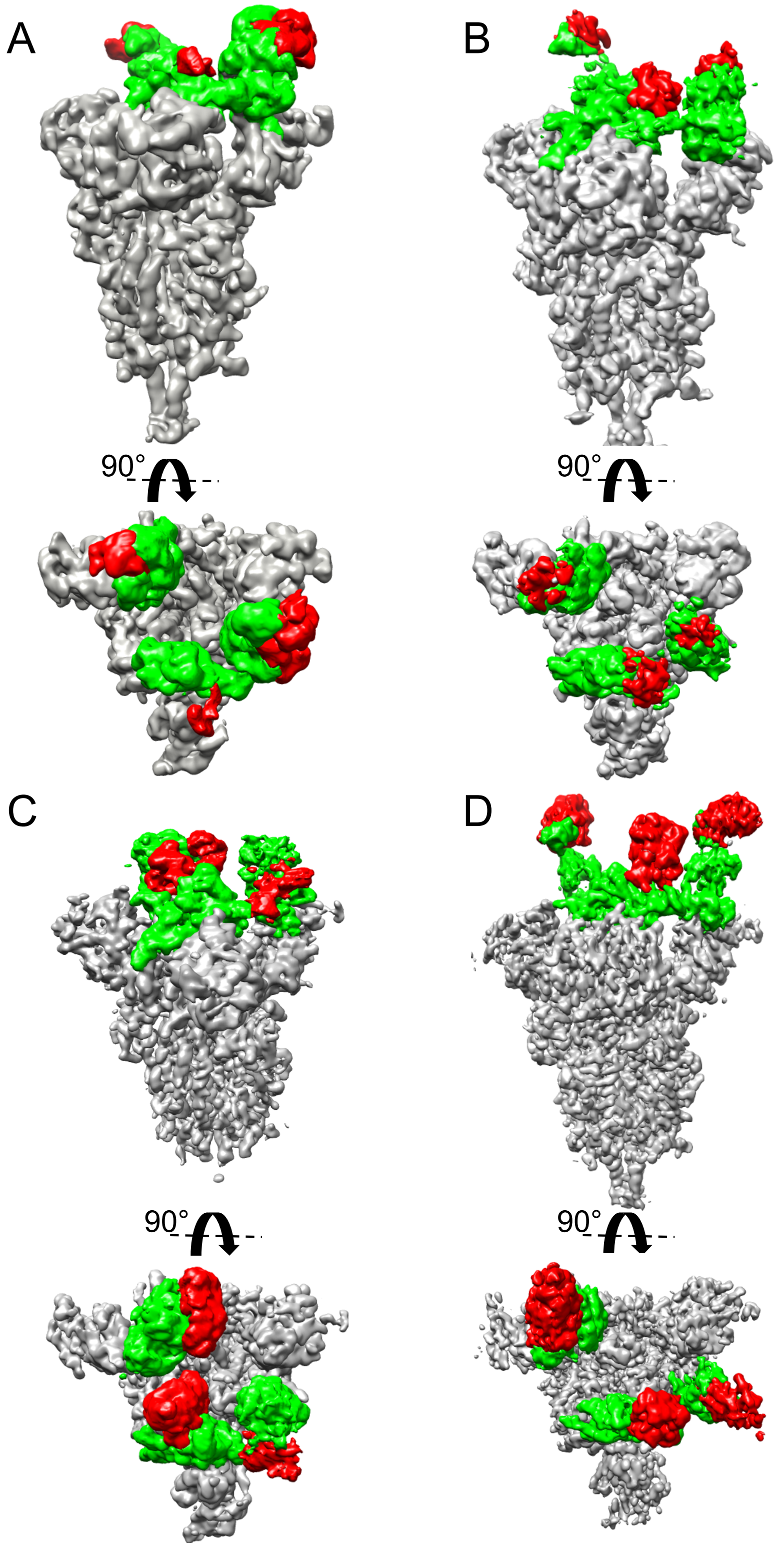

Figure S7. Cryo-EM structures of S-Nb complexes. Surface representations of the maps generated by the reconstruction of the trimeric S with bound 1.10 (**A**), 1.26 (**B**), 1.29 (**C**) or 2.15 (**D**) Nbs. Map regions corresponding to the Nbs are shown in red, the RBDs in green and the rest of the S in grey.

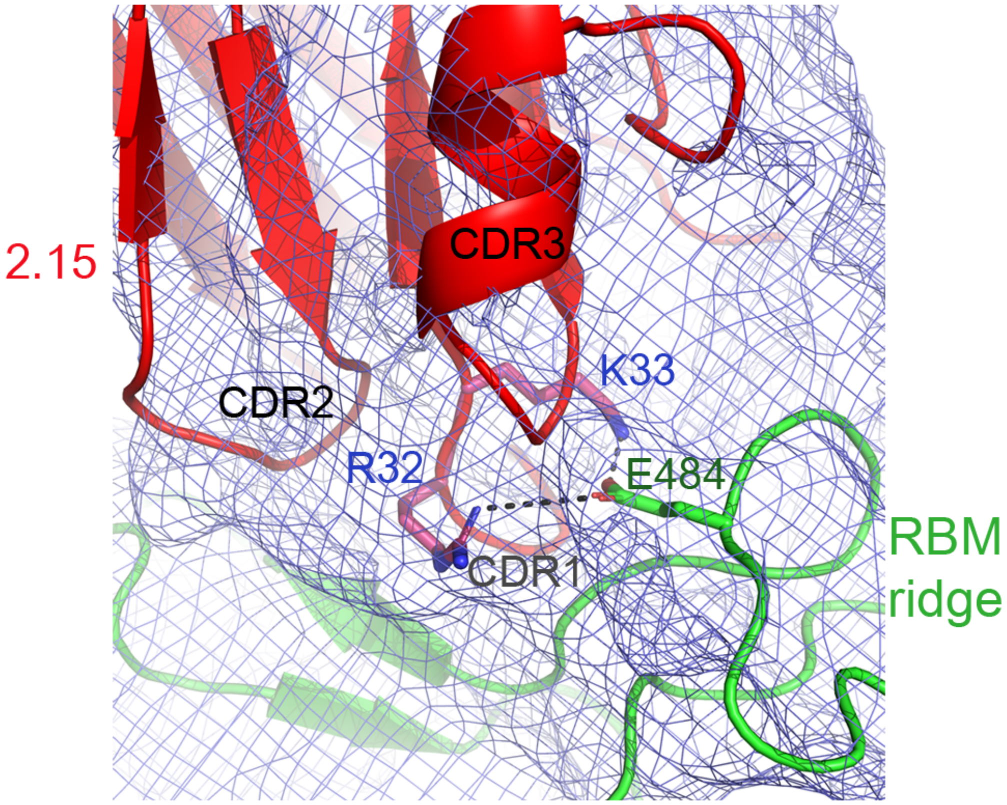

Figure S8. 2.15 Nb interaction with the SARS-CoV-2 RBM. Ribbon diagram of the Nb domain tip (red) that interacted with the RBM (green), as in Figure 6. The Nb CDR1, 2 ad 3 are labeled. Side chains of the Nb Arg32 and Lys33 in CDR1 that are closed to the RBD Glu484 are shown as sticks with nitrogens in blue and oxygens in red. Possible bonds between the basic Nb residues and Glu484 are shown as dashed black lines. Electron density displayed as a blue mesh countered at 1.5 sigma.

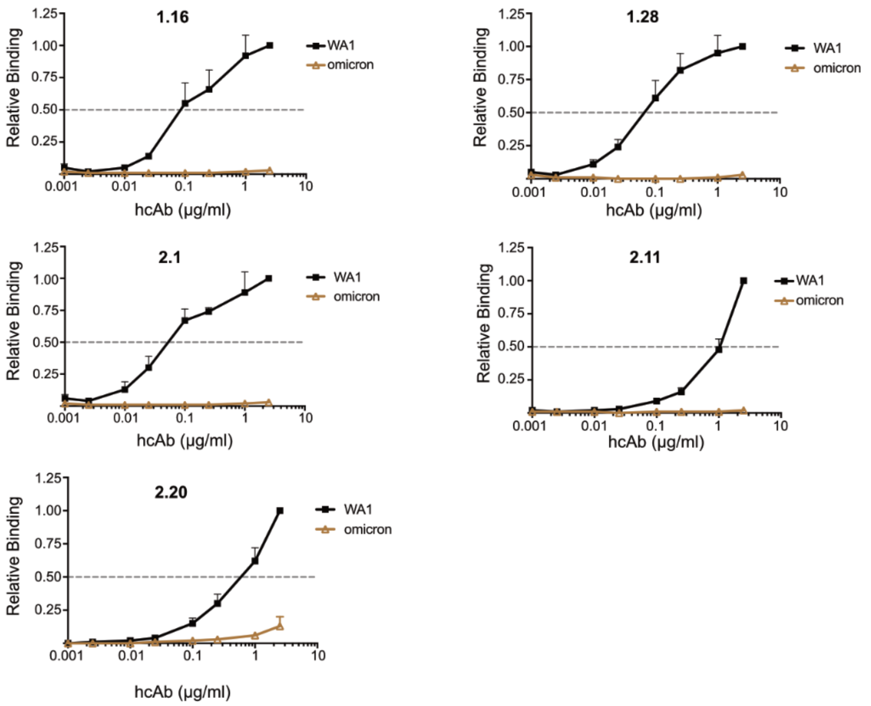

Figure S9. Binding of RBD WA1 and omicron by additional hcAbs of the panel. Group A 1.16, 1.28, 2.1, 2.11 and group B 2.20 (indicated on top of each graph) hcAbs binding to WA1 and omicron RBD-Fc proteins. The OD_{490} determined and normalized to the maximum binding signal with the WA1 protein as in Figure 7. The dotted line represents half of the maximum hcAb binding to WA1 RBD-Fc, used to determined EC_{50} values. Data are mean and standard deviation (SD) of three independent assays (n = 3).

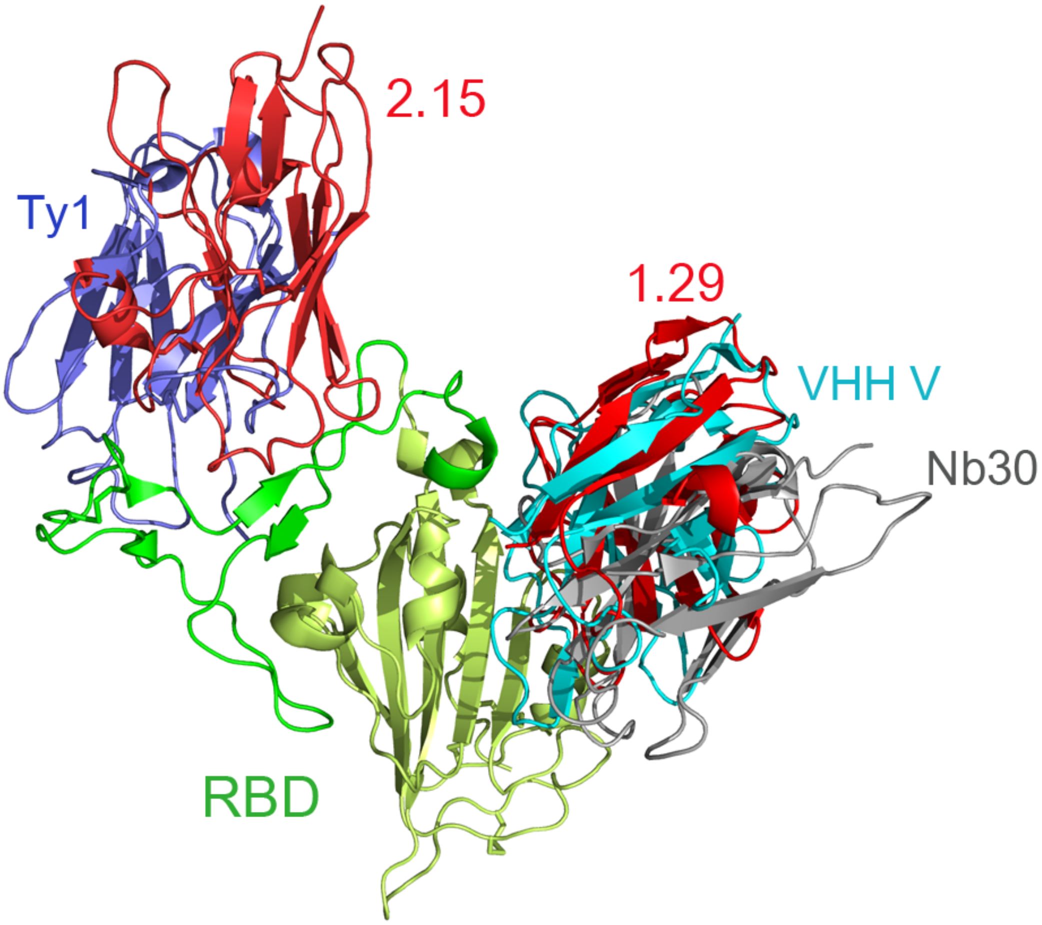

Figure S10. Nb binding to the SARS-CoV-2 RBD. Ribbon diagram of superimposed structures of the RBD in complex with Nbs 2.15, 1.29 (Figure 6), Ty1 (PDB ID 6zxn), VHH-V (PDB ID 7b17) or Nb30 (PDB ID 7MY2). Structures were superimposed based on the RBD.
